# Diagnosis and Treatment of Peripheral and Cranial Nerve Tumors with Expert Recommendations: An EUropean Network for RAre CANcers (EURACAN) Initiative

**DOI:** 10.3390/cancers15071930

**Published:** 2023-03-23

**Authors:** Alessia Pellerino, Robert M. Verdijk, Lucia Nichelli, Nicolaus H. Andratschke, Ahmed Idbaih, Roland Goldbrunner

**Affiliations:** 1 Division of Neuro-Oncology, Department of Neuroscience “Rita Levi Montalcini”, University and City of Health and Science Hospital, 10126 Turin, Italy; 2 Department of Pathology, Section Ophthalmic Pathology, Erasmus MC University Medical Center Rotterdam, 3015 Rotterdam, The Netherlands; 3 Department of Pathology, Leiden University Medical Center, 2333 Leiden, The Netherlands; 4 Department of Neuroradiology, Sorbonne Université, 75005 Paris, France; 5 Assistance Publique-Hôpitaux de Paris, 75610 Paris, France; 6 Groupe Hospitalier Pitié-Salpêtrière-Charles Foix, 75013 Paris, France; 7 Department of Radiation Oncology, University Hospital Zurich, University of Zurich, 8006 Zurich, Switzerland; 8 AP-HP, Hôpitaux Universitaires La Pitié Salpêtrière-Charles Foix, Sorbonne Université, 75005 Paris, France; 9 Inserm, CNRS, UMR S 1127, Institut du Cerveau-Paris Brain Institute, 75013 Paris, France; 10 ICM, Service de Neurologie 2-Mazarin, 75013 Paris, France; 11 Center for Neurosurgery, Department of General Neurosurgery, University of Cologne, 50923 Cologne, Germany

**Keywords:** schwannoma, neurofibroma, plexiform neurofibroma, perineurioma, hybrid nerve sheath tumor, malignant peripheral nerve sheath tumor, cauda equine neuroendocrine tumor, MEK inhibitors, mTOR inhibitors

## Abstract

**Simple Summary:**

The EUropean Network for RAre CANcers (EURACAN) Task Force on Ultrarare Brain Tumors (domain 10, subdomain 10) has reviewed the evidence of diagnostic and therapeutic interventions and drawn recommendations on peripheral and cranial nerve sheath tumors. The authors have provided an extensive revision of the clinical features, anatomical location, histological and molecular markers, and peculiar imaging findings of such unique entities, as well as some recommendations of local (e.g., surgery and radiotherapy) and systemic therapies (traditional chemotherapy and targeted agents) when feasible.

**Abstract:**

The 2021 WHO classification of the CNS Tumors identifies as “Peripheral nerve sheath tumors” (PNST) some entities with specific clinical and anatomical characteristics, histological and molecular markers, imaging findings, and aggressiveness. The Task Force has reviewed the evidence of diagnostic and therapeutic interventions, which is particularly low due to the rarity, and drawn recommendations accordingly. Tumor diagnosis is primarily based on hematoxylin and eosin-stained sections and immunohistochemistry. Molecular analysis is not essential to establish the histological nature of these tumors, although genetic analyses on DNA extracted from PNST (neurofibromas/schwannomas) is required to diagnose mosaic forms of NF1 and SPS. MRI is the gold-standard to delineate the extension with respect to adjacent structures. Gross-total resection is the first choice, and can be curative in benign lesions; however, the extent of resection must be balanced with preservation of nerve functioning. Radiotherapy can be omitted in benign tumors after complete resection and in NF-related tumors, due to the theoretic risk of secondary malignancies in a tumor-suppressor syndrome. Systemic therapy should be considered in incomplete resected plexiform neurofibromas/MPNSTs. MEK inhibitor selumetinib can be used in NF1 children ≥2 years with inoperable/symptomatic plexiform neurofibromas, while anthracycline-based treatment is the first choice for unresectable/locally advanced/metastatic MPNST. Clinical trials on other MEK1-2 inhibitors alone or in combination with mTOR inhibitors are under investigation in plexiform neurofibromas and MPNST, respectively.

## 1. Introduction

Cranial and peripheral nerve sheath tumors (PNST) comprise a heterogeneous group of soft tissue tumors. Most arise from classic peripheral nervous system elements (Schwann cells and perineurial cells), while others involve specialized neuroendocrine cells of the sympathetic and parasympathetic nervous system (e.g., cauda equina neuroendocrine tumors, previously known as “CNS paragangliomas”). The World Health Organization (WHO) classification of the Central Nervous System (CNS) Tumors of 2021 and the 2020 WHO Classification of Soft Tissue and Bone Tumors include benign and malignant tumors, such as schwannoma, neurofibroma, plexiform neurofibroma (PN), perineurioma, hybrid nerve sheath tumor (HNST), malignant peripheral nerve sheath tumor (MPNST), epithelioid MPNST or malignant melanotic nerve sheath tumor (MMNST), and cauda equine neuroendocrine tumor [1,2]. All these entities may arise along the craniospinal axis and be encountered either sporadically or as part of neurocutaneous syndromes, including neurofibromatosis type 1 (NF1), schwannoma predisposition syndromes (SPS), and Carney complex. Overall, PNST account for 12% and 8% of all benign and malignant soft tissue tumor neoplasms, respectively [3]. Interestingly, the WHO Classifications recognize some ultrarare and genetically unique entities, where the diagnosis may be challenging and based on a combination of clinical features, anatomical location, typical histological and molecular markers, and peculiar imaging findings.

## 2. Methods

The EUropean Network for RAre CANcers (EURACAN) established a multidisciplinary task force to develop expert recommendations on “peripheral and cranial nerve sheath tumors”. The task force reviewed the available English literature until September 2022, classified the scientific evidence into classes I–IV, and developed recommendations at levels A–C according to the European Federation of the Neurological Societies Guidelines [4]. In case sufficient evidence for recommendations was not available, the task force delivered advice as a good practice point or expert opinion.

## 3. Epidemiology and Clinical Features

Schwannomas are benign nerve sheath tumors composed of differentiated neoplastic Schwann cells. Up to 90% of schwannomas are solitary and sporadic, and affect people of all ages, with a peak incidence in the fourth to sixth decades of life. An increased incidence of schwannomas after prior irradiation has been reported [4]. A common presentation consists in an asymptomatic lesion of the skin or subcutaneous tissue of the head and neck, or along the flexor surfaces of the extremities. Spinal intradural extramedullary location is also frequent in schwannomas, that grow through neural foramina, causing radicular pain or sensory or, less frequently, motor symptoms. Spinal intramedullary and intracranial parenchymal schwannomas are rare [5], as well as the involvement of abdominal viscera (e.g., gastrointestinal tract) or bone [6]. Multiple paraspinal schwannomas are typical of neurofibromatosis type 2 (NF2). A frequent cranial location is the vestibular compartment of the eight cranial nerves. Vestibular schwannomas represent the third most common intracranial non-malignant tumor entity and comprise over 80% of tumors in the cerebellopontine angle [7]. Bilateral involvement is a criterion for NF2 [8]. Most of patients report unilateral hearing loss (94%) and tinnitus (83%), while vestibular symptoms, such as vertigo and unsteadiness, range from 17% to 75% of patients [9]. Trigeminal and facial neuropathies, brainstem compression, and hydrocephalus may occur in case of large cerebellopontine angle tumors. Schwannomas do not usually recur if treated by gross total resection. Malignant transformation is exceptionally rare, and few case reports have reported a transformation in epithelioid MPNST [10,11,12].

Neurofibroma is a frequent benign peripheral nerve sheath tumor (5.3% of all benign soft tissue tumors), and appears as a soft, skin-colored papule or small subcutaneous nodule. The most common site is the skin, with predominant dermal involvement; less frequently, the tumor involves medium-sized nerves, a nerve plexus, a major nerve trunk, or spinal nerve roots. Bilateral and/or multiple spinal root neurofibromas are typical of NF1, while cranial nerve neurofibromas are anectodical [13]. Cutaneous neurofibromas are usually asymptomatic, rarely painful, and the most common chief complaint is the cosmetic appearance [14]. Motor or sensory symptoms may occur when neurofibromas are in a deep location according with the distribution of the affected nerve. Neurofibroma can be classified into three types, such as localized or solitary, diffuse, and PN. Localized neurofibromas occur as polypoid lesions without any anatomical preference, while diffuse neurofibromas appear as plaque-like lesions mainly in the head and neck region. PN are large, massive lesions with “bag of worms” aspects, close to large spinal roots of the shoulder or pelvic girdle in adult population [15], while craniofacial site with disfigurement (35%), or location along limbs (19%), in association with pain and impairment of function, are typical of childhood [16]. The presence of multiple neurofibromas or PN is strongly suggestive of NF1, especially when associated with typical findings (see Section 4 “Genetic tumor syndromes correlated with cranial and peripheral nerve sheath tumors”). PN with progressive growth in adolescents and young adults may be considered as premalignant lesions and called atypical neurofibromatous neoplasms of uncertain biological potential (ANNUBP), which need careful surveillance to prevent malignant degeneration [17]. In general, PN carries an increased risk of malignant transformation. This holds true for NF1 patients with a lifetime risk increase of 8–13% to develop a malignant peripheral nerve sheath tumor (MPNST) [18], which is the most frequent cause of death in NF1 patients.

MPNST are rare aggressive tumors with an estimated incidence of 1.46 per 1,000,000 individuals [19] accounting for 2–10% of all soft tissue sarcomas [20]. Perineural MPNST are the most frequent form (93–95%), while epithelioid MPNST are particularly rare (~5% of all cases). Typically, MPNST arise from a pre-existing benign nerve sheath tumor, or deep-seated PN, or large intraneural neurofibroma, or ANNUBP in 8–13% of patients with NF1 [21], representing almost 50% of all MPNST cases. Another 40% of MPNST occur sporadically, and 5% following radiotherapy [22]. Patients with MPNST commonly are 20- to 50-year-olds and display large masses, primarily located along extremities, trunk, head, and neck area [23], that may cause pain or other neuropathic symptoms [18]. MPNST have a significant risk to recur (40–65%) and metastasize (40–80%) [24,25], resulting in a poor prognosis, with 5-year overall survival (OS) following treatments of 30–60% [24,26,27].

Malignant melanotic nerve sheath tumors (MMNST) are rare aggressive neoplastic lesions with fewer than 200 cases reported thus far [28]. MMNST occur mainly in young adults with a median age of 22 when associated with Carney complex (hereditary condition associated with spotty skin pigmentation, myxomas, and hormone-producing glands tumors), while in patients with sporadic presentation MMNST tend to occur later (median age of 33 years) [28,29]. Of note, the association between MMNST and Carney complex is not clearly established, with some series reporting >50% of patients with MMNST and Carney complex, and other showing an association in ≤5% only [28,30,31]. Most of MMNST are solitary lesions, usually located within spinal or autonomic nerves close to the midline. However, some atypical locations, such as the gastrointestinal tract [32,33], mediastinum [34], bone, soft tissues, bronchus, liver, and skin [1], have been reported as well. Local symptoms include pain, sensory deficits, mass effect, and bone erosion in case of spinal nerve root tumors, while systemic symptoms, such as respiratory and liver failure, may be found in patients with metastatic disease. Although MMNST have been considered benign with a metastatic risk <15% in past years, more recent reports have described an aggressive behavior, with an increased local recurrence and metastatic rate in 26 to 44% of patients [28,31,35], thus suggesting that a long-term follow-up is required.

Perineuriomas are slowly progressive peripheral nerve tumors arising from perineural cells, representing approximately 1% of peripheral nerve tumors [36]. Most of them occur in major peripheral nerves and their branches, such as the sciatic nerve (42%), median nerve (13.5%), radial nerve (15.6%), and brachial plexus (12.2%). Uncommon locations are cranial nerves [37,38], lateral ventricle [39], oral cavity, skin, and mandible [40]. Most of patients present a focal, unilateral lesion, while only a few have been reported to present with bilateral (2.7%) or unilateral multifocal lesions (1.3%) [40]. Clinically, intraneural perineuriomas prevail in adolescence or early adulthood (median age of 14 years) and come to clinical attention nearly 2 years after initial onset of symptoms [41], while soft tissue perineuriomas peak in adult age (40–50 years) [1]. The main neurological symptom is a painless mononeuropathy with associated muscle atrophy and typical fusiform nerve enlargement according to the Perineurioma Diagnostic Criteria [42]. Sensory symptoms or pain are rare. Clinical worsening in neurological symptoms or malignant transformation have not been reported in case of observation or conservative approach thus far [36]. Interestingly, limb undergrowth or hand/foot discrepancy have been reported in 25% of patients with perineuriomas, especially in those with proximal lesions in the lower extremity [43]. Some patients with NF type 1 and 2 may develop perineuriomas, but it is unknown whether this is a true association [44,45,46,47].

Hybrid nerve sheath tumors (HNST) are an extremely rare entity, characterized by hybrid lesions with mixed elements of neurofibroma, schwannoma, and/or perineurioma. Typically, HNST consists of a painless mass in subcutaneous tissue or dermis [48]; when large peripheral nerves or spinal nerves are involved, the tumor may be associated with pain or neurological impairment. The most common site of HNST are the fingers [49], but some rare cases arise from cranial nerves [50,51,52]. Hybrid schwannomas/perineuriomas are sporadic [52], while hybrid neurofibroma/schwannomas are strongly associated with NF1 and 2, and schwannomatosis [53]. Hybrid neurofibroma/schwannoma is the most represented morphology (71%) in schwannomatosis [53,54], while hybrid neurofibroma/perineurioma has been reported in association with NF1 [55,56]. Overall, HNST are benign tumors with a rare propensity to recur locally.

Neuroendocrine tumors of the cauda equina are extremely rare, with 300 cases reported thus far, representing overall <3% of spinal tumors [57]. Cauda equina neuroendocrine tumors usually affect adults, with a peak of incidence in the fourth–sixth decades of life, with a median age of 46 years (range 6–85) [58]. Notably, when neuroendocrine tumors affect the spinal cord, the cauda equina represents the most frequent location, but uncommon locations in the cervical and thoracic regions have been reported [59,60,61]. Most of cauda equina neuroendocrine tumors are considered sporadic, with slow-growing behavior, and neurological symptoms correlated to the site of presentation, including low back and/or radicular pain, numbness, paraparesis, sphincter symptoms, or complete cauda equina syndrome [62,63]. Extracranial metastases to the bone have been reported only in one case [64], while CSF dissemination may occur occasionally [65,66,67] with signs of increased intracranial pressure with papilledema [63] and increased CSF proteinorrachia [68]. Some rare neuroendocrine tumors of the cauda equina can produce circulating catecholamines, leading to episodic or sustained hypertension crisis, palpitations, diaphoresis, headache [62], or subarachnoid hemorrhage [68].

The Table 1 summarizes epidemiologic and clinical features of cranial and peripheral nerve tumors.

## 4. Genetic Tumor Syndromes Correlated with Cranial and Peripheral Nerve Sheath Tumors

Some genetic tumour syndromes predispose to the development of central and PNST, such as NF1 and SPS. NF1 is an autosomal dominant disorder with a complete penetrance due to a germline pathogenic variant in the NF1 gene on 17q11.2, which is a down-regulator of the oncogene Ras. The prevalence of the disease is of 1/2000 to 1/4000 persons without any difference in incidence by sex or race. Phenotype may be heterogenous among members of the same family [69]. Genetic mosaicism, resulting from somatic pathogenic variants in the NF1 gene after fertilization, represents 30–50% of “de novo” cases of NF1 and may cause anatomically limited or clinical attenuated phenotype. Several non-neoplastic manifestations may be present, including café au lait macules, skeletal abnormalities (scoliosis—often associated with paraspinal tumors, pseudarthrosis, early osteoporosis, long-bone dysplasia), short stature, vascular and congenital cardiac anomalies (renal artery stenosis, moyamoya syndrome, aneurysms), and macrocephaly. Learning disability and attention deficit/hyperactivity disorders occur in 50% or more of patients with NF1. PNST associated with NF1 are cutaneous neurofibromas (99%), PN (40–50%), MPNST (8–13%) in adult patients with NF1. Other tumors associated with NF1 are optic pathway gliomas, with the greatest risk prior to age of 6 years (15–20%), breast cancers, with greatest increase in risk between 30 and 40 years of age, pheochromocytomas (1–5%), GIST, and glomus tumors [70]. Legius et al. [71] have revised the major developments in genetics, ophthalmology, dermatology, neuroimaging, and provided an update on diagnostic criteria for NF1. Molecular diagnosis of NF1 is confirmed when an NF1 pathogenic variant is identified in an individual/fetus having either one or more of the other clinical diagnostic criteria. In fact, the identification of an NF1 variant alone does not allow a diagnosis of NF1, and requires further clinical and genetic evaluation [71]. Dosage analysis for the identification of copy-number variants, and DNA-based sequencing, can identify a pathogenic variant in approximately 90% of classic NF1 patients (e.g., with pigmentary features, as well as neurofibromas). Detection rate and specificity are increased to 95–97% when an RNA-based sequencing, in addition to dosage analysis, is applied [72]. Mosaicism is confirmed when a patient with features of NF1 carries a heterozygous NF1 pathogenic variant with a variant allele fraction (VAF) < 50% in an unaffected tissue (e.g., blood). Mosaicism is also confirmed if an identical pathogenic NF1 variant is identified in two or more anatomically unrelated lesions in the absence of this pathogenic variant in unaffected tissue such as blood [71]. In 2023, the European Reference Network on Genetic Tumour Risk Syndromes (ERN GENTURIS) has provided a tumour surveillance guideline for patients with NF1, with the aim to detect tumors before they become symptomatic and for interventions to have a better chance of being curative or prevent functional impairment [73].

Neurofibromatosis type 2 (NF2) and schwannomatosis (SWN) are genetically distinct tumor predisposition syndromes with overlapping phenotypes, of whom diagnostic criteria have been recently updated. A diagnosis of NF2 can be made in case of bilateral vestibular schwannomas, presence of an identical NF2 pathogenic variant in at least two anatomically distinct NF-2-related tumors (e.g., schwannoma, ependymoma, meningioma) or when two major criteria or one major plus two minor criteria are met (major criteria: unilateral vestibular schwannoma, first-degree relative other than sibling with NF2, ≥2 meningiomas, NF2 pathogenic variant in unaffected tissue—if VAF < 50%, the diagnosis is mosaic NF2; minor criteria: more than one type of tumors, including ependymoma, meningioma, or schwannoma, juvenile subcapsula or cortical cataract, retinal hamartoma, epiretinal membrane in a person < 40 years) [74]. Genetic analysis uses next-generation sequencing plus multiplex ligation-dependent probe amplification for the detection from 1 exon to multiexon copy number changes and high resolution karyotyping for the identification of chromosomal rearrangements in the NF2 locus with a germline detection rate of 96% in the second generation of families with typical NF2 [75]. The germline detection rate decreases to 60% in mosaic NF2 [76].

SWN is a clinical distinct entity as a result of germline NF2 (22q-related schwannomatosis), or SMARCB1 (SMARCB1-related schwannomatosis), or LZTR1 variant (LZTR1-related schwannomatosis), which are located centromeric to NF2 on chromosome 22 [74,77]. Germline SMARCB1 or LZTR1 pathogenic variant account for 70–80% of familiar SWN, but 30% only of sporadic cases [77]. Major criteria for the diagnosis of SWN are at least one pathologically confirmed schwannoma or HNST and an SMARCB1 or LZTRK1 pathogenic variant in unaffected tissue (e.g., blood) or a shared SMARCB1 or LTRK1 pathogenic variant in two schwannomas or HNST [74]. As several tumor types and clinical features are shared by NF2 and SWN, a patient suspected for one of these SPS should undergo comprehensive genetic testing to achieve the correct diagnosis.

## 5. Pathology and Molecular Markers

Tumor diagnosis for most of cranial and paraspinal nerve sheath tumors is still primarily based on hematoxylin and eosin (H&E)-stained sections and some additional techniques, including immunohistochemistry. Molecular testing generally is not required for this type of tumor but may be of help in the distinction of low-grade MPNST from cellular or atypical neurofibroma. However, mutation analysis of PNST may be required to diagnose mosaic forms of NF1, NF2, and SPS through the identification of the same mutation in at least two independent tumors as this is often the only way to prove a mosaicism. A summary of the essential diagnostic criteria for the tumors covered in this guideline is presented in Table 2.

Schwannoma. Conventional schwannoma is an encapsulated tumor comprised almost exclusively of neoplastic Schwann cells arranged in an alternating pattern of hypercellular Antoni A areas and hypocellular Antoni B areas. Nuclear palisading may be present, and Verocay bodies may be seen. The stroma can show hyalinized blood vessel walls and foamy macrophages. Rare histological variants include cellular, plexiform, microcystic, reticular, and epithelioid schwannomas. Tumors of the eighth cranial nerve are unencapsulated and predominantly show Antoni A tissue. Ancient schwannoma differs from conventional schwannoma only by its presence of scattered atypical to bizarre-appearing nuclei. A malignant change of schwannoma is exceptionally rare [1,2]. The tumour cells are diffusely and strongly positive for S100 protein, with both nuclear and cytoplasmic staining and show uniform nuclear positivity for SOX10 [78,79]. Loss of SMARCB1 (INI1) expression is found in epithelioid schwannoma, or a mosaic pattern of SMARCB1 (INI1) expression indicates syndrome-associated schwannoma [80,81]. Schwannomas may exhibit complete or partial loss of chromosome 22. Despite frequent NF2 alterations in schwannomas, this is not specific, and a pathognomonic molecular signature has not been found. However, schwannomas exhibit a distinct DNA methylation pattern [1,2].

Neurofibroma and plexiform neurofibroma. Neurofibromas show a diffuse proliferation of Schwann cells, nerve sheath fibroblasts, and less markedly axons permeating the lesion in a haphazard fashion in a myxoid and collagenous stroma. Furthermore, neurofibromas show a disperse positive staining for S-100 of only a portion of the tumor cells. An increased cellularity in the absence of other features may be seen in cellular neurofibromas. Mitotic figures are not usually present and may denote an atypical neurofibroma (AN) or atypical neurofibromatous neoplasm of uncertain biological potential (ANNUBP) in the setting of NF1. Malignant transformation requires a triad of increased cellularity, nuclear pleomorphism, and increased mitotic figures. The current WHO classification provides exact criteria for NF vs. ANNUBP and MPNST [1,2]. Localized cutaneous neurofibromas are consistently benign, while PN, ANNUBP, and solitary intraneural neurofibromas arising in sizeable nerves can be precursor lesions of MPNST. A biallelic genetic inactivation of the tumor suppressor gene NF1 is almost invariably present in NF [82]. Histological features of AN/ANNUBP are strongly associated with deletions of the CDKN2A/CDKN2B locus, which may be demonstrated immunohistochemically by p16 loss [2]. Inactivation of SUZ12 or EED, leading to H3K27 trimethylation loss, denotes progression to MPNST [2].

Perineurioma. Perineurioma is composed of spindle cells with wavy or tapering nuclei, indistinct nucleoli, and bipolar cytoplasmic processes arranged in a storiform or whorled growth pattern. The intraneural lesions form typical pseudo-onion bulbs. Tumor cell stain is variably positive for EMA, claudin-1, and GLUT1. CD34 is expressed in 60% of cases. The cells are negative for S100 and SOX10 [1,2]. Intraneural perineuriomas harbor missense mutations in TRAF7 [83], whereas soft tissue perineuriomas commonly show deletions of chromosome 22q (NF2) and deletions of chromosome 17q11 (NF1) [84], as well as chromosome 2p deletions or rearrangements or deletions of chromosome 10q (sclerosing variant) [85].

Hybrid nerve sheath tumors. HNST are benign PNST with combined features of more than one conventional type (neurofibroma, schwannoma, perineurioma) [1,2]. The molecular features of the dual differentiation, which is typical of hybrid tumors, are largely unknown. Activating ERBB2 mutations have been identified in a subset of neurofibroma/schwannoma hybrid tumors [86].

Malignant peripheral nerve sheath tumor (MPNST). Morphologic criterion for the diagnosis of MPNST is the presence of spindle cells with indistinct cytoplasmic margins and wavy or S-shaped nuclei, that are arranged in fascicles with alternating cellular and myxoid areas. Brisk mitotic activity is usually present. Rare dispersed, single-tumor cells stain positive for S100 in about 65% of tumors [1,2]. Most of MPNST show homozygous inactivation of NF1 and CDKN2A and/or CDKN2B. Inactivation of the core components of PRC2, SUZ12, or EED is the most important molecular marker for MPNST [87,88,89,90]. This leads to loss of H3K27 trimethylation, which may be demonstrated by immunohistochemistry [91,92]. Other complex genomic rearrangements are commonly seen.

Epithelioid malignant peripheral nerve sheath tumor. Epithelioid MPNST are composed of epithelioid cells with abundant eosinophilic cytoplasm, sometimes embedded in an abundant myxoid or hyalinized stroma. Epithelioid MPNST show a strong and diffuse staining for S100 and SOX10, with retained H3K27 trimethylation and loss of SMARCB1 expression [1,2]. Epithelioid MPNST are driven by SMARCB1 gene inactivation in about 80% of the cases [81].

Malignant melanotic nerve sheath tumor. The tumor consists of plump spindle cells arranged in short fascicles or sheets. Vague palisading or whorled structures may be observed. Tumor cells have eosinophilic to amphophilic cytoplasm with round to ovoid nuclei showing nuclear grooves and pseudoinclusions and usually small nucleoli. Melanin pigment may be coarsely clumped or finely granular and varies from area to area. Mitoses and necrosis can be present, but this is not a grading criterium. The behavior of MMNST is difficult to predict and metastases can occur in the absence of morphologically malignant features. Psammoma bodies are present in about 50% of cases (psammomatous MMNST). MMNSTs strongly express S100, SOX10, and multiple melanocytic markers, such as HMB45, melan-A, and tyrosinase [1,2]. PRKAR1A mutations are present in the majority of cases [28,31].

Cauda equina neuroendocrine tumor (previously paraganglioma). Microscopic appearance is that of a highly cellular tumor of round to polygonal cells with finely granular eosinophilic cytoplasm. A typical nested pattern may be highlighted by reticulin staining and immunohistochemistry for S-100, which shows the sustentacular cells around the nests. The tumor cells are positive for chromogranin and synaptophysin and may be positive for keratins [1]. The molecular alterations in cauda equina neuroendocrine tumors are unknown, although overexpression of HOXB13 [93] and typical methylation profile and CNV have been described [94,95]. Cauda equina neuroendocrine tumor is not associated with (germline) SDH subunit mutations. The combination of RB1 loss of function and TP53 mutations should alert for metastatic neuroendocrine tumors.

## 6. Imaging

Imaging is used to orient the diagnosis, delineate lesion margins and involved structures, and monitor central and peripheral nerve sheath tumors. Ultrasounds should be the first-line imaging procedure in the presence of a superficial musculoskeletal soft-tissue lesion. This affordable, radiation-free technique allows an initial exclusion of mimicking pathologies with high diagnostic accuracy [96,97] and can guide percutaneous biopsy with a reasonably low complication rate [98,99]. Computerized tomography (CT) should not be used to characterize neurogenic lesions, as it displays low contrast resolution in soft tissue structures and exposes patients to ionizing radiation. However, CT can detect bone remodeling and/or erosion [100], hemorrhagic transformation in emergency settings [101], and calcifications [102].

Magnetic resonance imaging (MRI) is the gold standard imaging modality to characterize soft tissue lesions [103,104] and delineate their extension to adjacent structures (especially neurovascular structures and muscular fascia). MRI can non-invasively suggest the neurogenic origin of a soft tissue mass and may help to distinguish between benign and malignant variants. However, MRI is limited in the distinction between schwannoma and neurofibroma [105], as well as between schwannoma and MPNST [106,107]. The MR-imaging-based Neuropathy Score Reporting and Data System (NS-RADS) is a recently developed framework to standardize MRI reporting in peripheral neuropathy [108,109]. It is divided into classes (letters) and subclasses (Arabic numbers), which correspond to different underlying disorders and severity grades or extents, respectively, including muscle denervation changes. Neural and/or perineural neoplasia is labeled with the letter N and divided into four subclasses from N1 to N4. N1 and N2 denote a definitely or probably benign condition, N3 a probably malignant disorder, and N4 a recurrent tumor (highly suggestive of malignancy). MRI optimal protocol for peripheral nerve sheath tumor pathology has also been recently reviewed by the NS-RADS group [109].

Positron emission tomography (PET) combined with CT is useful in detecting malignant transformation of a PNST, and identifying systemic metastases, and thus guiding biopsy [110]. F18 fluorodeoxyglucose (FDG) is the most used PET tracer. Early and delayed (60–90 min and 240 min, respectively) FDG-PET/CT scans diagnose NF1-associated MPNST with high (~90%) sensitivity and specificity values [106]. Benign lesions display low (<2–3) maximum standardized uptake value (SUVmax) on early and delayed FDG-PET scans, while MPNST show increased (>3–4) values. PET/MRI has been suggested as a feasible alternative to PET/CT in patients with NF1 when screening for presence of MPNST. The main benefit is to avoid the exposure to ionizing radiations from CT with similar accuracy (100%) of PET/MRI to detect MPNST as compared with PET/CT [111,112]. 11C-methionine tracer increases PET specificity in ambiguous lesions [113].

MRI features of a neurogenic tumor. The direct continuity between a nerve and a lesion, resembling a tail coming off the lesion, is called a tail sign [106,107]. This feature is almost pathognomonic of a PNST, particularly when seen along the long axis of a lesion and when a large nerve is involved. However, MRI does not discriminate between benign and malignant conditions. The tail sign is more commonly located in the central part of the lesion in neurofibromas, while it is more eccentrically located in schwannomas. A fusiform or round shape is also suggestive of a neurogenic tumor. Muscle denervation changes, including edematous changes, fatty infiltration, and/or atrophy of the innervated muscle, also strongly suggest the diagnosis [107,108].

MRI features of benign PNST. Several findings have been described in benign PNST, including a tail sign, fusiform shape, well-defined margins, a target sign (a low or intermediate signal intensity in the center of the lesion, surrounded by a peripheral hyperintense ring), a fascicular sign (various thin ring-like structures), and a split fat sign (a fat rim that separates the tumor from the surrounding tissue) [107,114,115]. Wide window settings allow a better detection of most of these imaging characteristics. Lesions are usually T1 hypointense, T2 hyperintense, and display a significant Gadolinium enhancement, which is delayed on dynamic contrast-enhanced imaging. Fat suppressed T2 and T1 sequences after contrast injection are often the most helpful to show the lesion. Localized benign tumors are usually well-defined, while diffuse neurofibromas are often poorly defined. Small neurofibromas show a homogenous or targetoid enhancement, while larger lesions have a more heterogeneous enhancement. PN display a multinodular/fascicular/network-like appearance that may involve multiple nerve branches, sometimes resembling a “bag of worms”. Based on MRI locations, PN are classified as superficial or deep, although a combination of these two entities can exist. Superficial lesions are more common, respect cutaneous/subcutaneous planes, and present a diffuse, ill-defined reticular morphology, that can be misdiagnosed as venous malformation [115]. Deep neurofibromas do not involve skin or subcutaneous tissues and can exhibit a target-like appearance on T2-weighted images.

**Figure 1:**
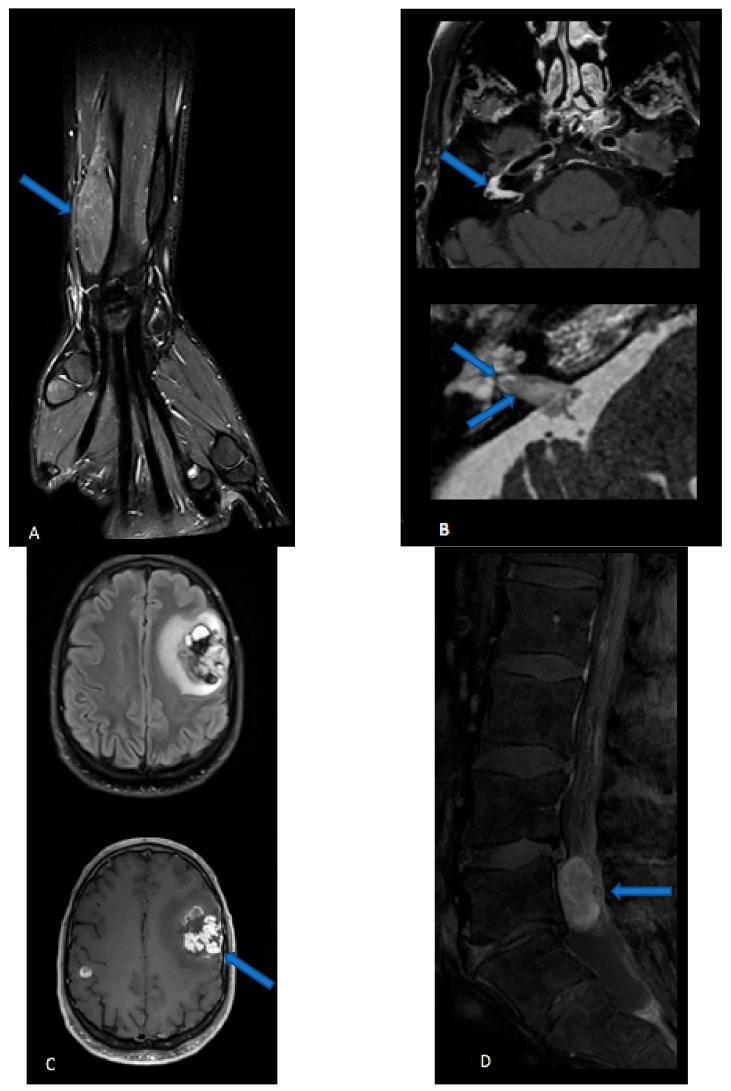
(**A**) Perineurioma: coronal three-dimensional (3D) proton density sequence of the wrist after fat suppression displays a fusiform enlargement of the radial nerve. (**B**) Malignant peripheral nerve sheath tumor: Three-dimensional T1-weighted spin echo sequence after contrast injection and fat suppression (upper image) shows a contrast-enhanced lesion in the right internal auditory canal and geniculate ganglion. High-resolution 3D T2-weighted sequence (lower image) reveals intra-cochlear extension of the tissue mass. (**C**) Malignant intracerebral nerve sheath tumor: 3D FLAIR sequence in the axial plane (upper image) and 3D T1-weighted spin echo sequence after contrast injection shows two intra-axials, enhancing lesions. The biggest lesion, located in the left superior frontal region, is highly heterogeneous, with hemorrhagic and cystic components, and is surrounded by FLAIR hyperintensity. The second, smaller lesion of the right hemisphere is hardly visible on the FLAIR image while it strongly enhances after contrast administration. (**D**) Cauda equine neuroendocrine tumor: sagittal T1-weighted sequence after contrast injection and fat suppression of the lumbar spine displays a well-defined intradural extramedullary contrast, enhancing lesions.

Perineuriomas are seen as a gradual, uniform nerve enlargement, followed by a gradual narrowing (Figure 1A). Individual fascicles are uniformly enlarged, displaying a characteristic “honeycombing pattern” [106]. Increased apparent diffusion coefficient (ADC) values (>1.1 × 10^−3^ mm^2^/s) and functional anisotropy (FA) values are also seen [116]. On MRI spectroscopy, the trimethylamine fraction is usually low (<50%) [117]. Bone destruction is not a specific feature in the distinction between benign and MPNST [118]. The radiological features of HNST are lacking: a single case report described multiple nerve sheath lesions with a bright signal on STIR sequence, peripheral enhancement, and large avascular regions [119].

MRI features of MPNST (Figure 1B). If typical findings of a benign PNST are absent, an MPNST should be suspected. The presence of infiltrative margins, peritumoral edema and/or necrosis, intra- or peritumoral hemorrhage, an irregular or round shape, a size greater than 5 cm, and a heterogeneous enhancement all suggest a malignant tumor [120]. ADC values are low (<1.1 *×* 10^−3^ mm^2^/s), nerve tracts are partially or completely disrupted in diffusion tensor studies, and trimethylamine fraction is high (greater than 50%) in MPNST. In the case of neurofibromatosis or in the follow-up of a known benign PNST, a rapid growth is also evocative of malignant degeneration [121]. Of note, it can be difficult to establish malignancy on imaging, and some radiological findings (heterogeneity, diffusion restriction) are less concerning in schwannoma in comparison to neurofibroma and plexiform neurofibroma. MPNSTs have a strong tendency to metastasize, especially in the spinal canal, and therefore spine imaging should be performed. In the neuroaxis, extra-axial MPNSTs are commonly separated from their intraparenchymal counterparts, which are termed malignant intracerebral nerve sheath tumors (MINST) (Figure 1C) [122,123]. MINST display nonspecific high-grade tumor features (high heterogeneity, variable necrotic, hemorrhagic, cystic and calcific components, irregular enhancement) and therefore are indistinguishable from a high-grade glial neoplasm or a solitary metastasis. They have been described in both infratentorial and supratentorial location, in the sellar and intraventricular region, and can be solitary or multiple and display variable mass effect [124]. Interestingly, in a single case report of a pathologically proven MINST, absence of creatine and N-acetyl aspartate resonance was seen on MR spectroscopy [125], suggesting a non-glial origin of the neoplastic lesion [126]. Extra-axial MPNST features are also not pathognomonic and can mimic those of meningioma [124].

MRI features of cauda equina neuroendocrine tumor (Figure 1D). In cauda equina neuroendocrine tumor, MRI shows non-specific findings of an intradural, extramedullary contrast-enhancing lesion. The tumor is often circumscribed, oval-shaped and encapsulated [127], with a variable attachment to the filum terminale [68], and usually separated from the nerve roots [62]. Cystic, fibrotic, hemorrhagic, and necrotic areas may be seen [128], and syringomyelia has been reported [120]. Erosion or scalloping of vertebral laminae and flattening of pedicles may occur, witnessing a slow-growing mass [128]. Associated spondylolisthesis and scoliosis may be present [62]. Prominent serpiginous flow voids have been suggested as major clues to the diagnosis of these highly vascular lesions. Cauda equina neuroendocrine tumors are also a potential cause of brain and spinal cord superficial siderosis [129]. Spine or brain metastases are rare [130].

## 7. Surgery

The vast majority of PNST is benign and well-circumscribed; therefore, neurosurgical resection is the therapy of choice not only to gain tissue for the diagnosis but also to cure the tumor [131,132]. Principles of surgery in the different entities of tumors involving peripheral nerves, such as schwannoma, neurofibroma, perineurinoma, HNST, MPNST, and cauda equina neuroendocrine tumors, are similar, but might differ depending on tumor size, location, attachment to neighboring structures, and malignancy. Basically, total removal of the tumor is the goal of surgery. However, the extent of surgery must be balanced to preservation of nerve function. Complete resection can be achieved by intracapsular dissection of the tumor mass, preserving the attached functional nerve fibers [131,132,133]. Intraoperative electrophysiology, especially direct motor nerve stimulation, enables the neurosurgeon to detect functional nerve fibers, even if they are displaced by the tumor, and to monitor nerve function during surgery. Therefore, the use of intraoperative electrophysiological monitoring is regarded as mandatory [131,132]. Intraoperative high-resolution ultrasound might be helpful in identifying the tumor [134]. There are no prospective studies on the use of a microscope to visualize nerve fibers intraoperatively, but according to expert opinions, this is recommended as a good practice point. Surgery is effective in pain management [135], especially in sporadic tumors [131]. Rates of complete resection depends on the presence of a genetic disorder (schwannomatosis or neurofibromatosis). In case of NF2, resection rates up to 82% are reported [135], whereas in spontaneous tumors, up to 92.5% may be achieved [133]. The risk for permanent or temporary new neurological deficits in benign lesions might be up to 15.2% [136]. Recurrence rates in genetic syndromes, such as schwannomatosis or NF2, can be as high as almost 40% [131,136] after 5 years and 100% after 10 years [131]. In benign nerve tumors, there is a large number of case series showing that surgery allows significant pain reduction and long-term progression-free survival, depending on the presence of a genetic disorder [131,133,135,136]. Despite a lack of level I or II evidence, total or subtotal resection with preservation of nerve function is recommended as therapy of choice.

Cranial nerve tumors, such as vestibular schwannomas (VS), require a careful evaluation for surgery. Observing VS with serial MRI and audiological monitoring without any tumor-directed treatment is considered appropriate for incidental, asymptomatic VS [137]. However, approximately 50% of VS may be expected to grow over a 5-year period [138,139] with a risk of 50% to lose functional hearing during a 3–4-year period [140]. Treatment options are (complete or subtotal) resection or radiosurgery. In case of surgery, the risk of recurrence after gross-total resection should be balanced with the risk for facial nerve dysfunction and hearing impairment. In this regard, to improve the nerve functional preservation, intraoperative monitoring is mandatory for surgery of VS and should include somatosensoric evoked potentials, monitoring of the facial nerve comprising direct electrical stimulation and free-running, brainstem auditory evoked responses, and electromyography of the lower cranial nerves in case of a large lesion [141]. Different surgical approaches may be employed for VS, such as suboccipital retrosigmoid (retromastoid), or translabyrinthine, or middle fossa approach. Overall, there are no sufficient data supporting the superiority of any approach in terms of extent of resection and nerve function preservation [142], thus the surgical approach should be chosen upon hearing status, tumor characteristics, patient’s preferences, and surgeon’s expertise.

Similar to soft tissue sarcomas, survival of patients with MPNST clearly depends on extent of resection, as shown by several series [131,143,144]. Therefore, R0 resection, including a safety margin up to 2 cm, is recommended, if this is feasible and accepted by the patient. In cases of recurrent MPNST in extremities, even limb amputation might be necessary. The Table 3 summarizes the potential impact of surgery on outcome.

## 8. Radiotherapy

Radiotherapy is a central pillar in the multimodality treatment of soft tissue sarcomas and CNS tumors, also of mesenchymal origin like meningiomas and vestibular schwannomas. Depending on individual patient and tumor situation, it can be applied either as sole definitive treatment with curative intent or as pre- or post-operative adjunct to surgery to maximize local control in difficult-to-treat tumors or with palliative intent to alleviate symptomatic burden of the disease. The current standard of treatment planning and delivery is the use of multi-modality co-registered imaging for target volume definition, intensity modulated radiotherapy—either delivered as step-n-shoot or volumetric rotational radiotherapy—with integrated online image guidance. Currently, standard fractionation is mostly utilized with single fraction doses of 1.8–2.0 Gy up to total doses of 45–66 Gy, depending on the decision on pre- or post-operative radiotherapy. In case of definitive radiotherapy, stereotactic radiotherapy with single-fraction or multi-fraction radiosurgery has evolved as an alternative to conventional fractionated radiotherapy, especially for small volumes which are distant to critical organs where small safety margins can be applied. As there is no prospective (randomized) data available on the optimal treatment of CNS and peripheral nerve tumors, reliance on retrospective studies and case series, as well as on analogy assumptions from soft tissue sarcoma and CNS tumor management, is sensible.

Malignant nerve sheath tumors. Due to its aggressive nature and tendency for local relapse, radiotherapy has been considered for high-grade or large (>5 cm) tumors as well as after incomplete (R1 or R2) resection with a trend for improved local control [145], but was not associated with improved overall survival as suggested by a recent nation-wide registry analysis [146]. With the availability of the prospective study on non-rhabdomyosarcoma soft tissue sarcomas in patients under 30 years of age (COG study ARST0332; 11% of MPNST included), some guidance can be derived given the encouraging results, although generalization to older patients remains open [147]; only in patients with low-grade tumors and gross total resection (R0 and R1) radiotherapy had been omitted. In the remainder, radiotherapy was applied post-operatively in case of a high-grade histology and R1 resection or a tumor greater than 5 cm, and pre-operatively in case of unresectable tumors. In case of planned post-operative radiotherapy, 55.8 Gy in 1.8 Gy per fraction were applied. After neoadjuvant radiotherapy with 45 Gy in 1.8 Gy per fraction, final dose was determined by the resection status, and a boost of 10.8 Gy (R1 resection; cumulative dose 55.8 Gy) or 19.8 Gy (R2 resection; cumulative dose 64.8 Gy) was applied if necessary. Thus, in the current decision-making process, the above-mentioned risk factors for an increased risk for local recurrence and the overall prognosis should be considered individually when assessing the need for additional radiotherapy. As R0/1 resection remains one of the strongest predictors of overall survival, pre-operative radiotherapy should be strongly considered in unresectable cases to increase the potential of an R1 or even R0 resection.

Schwannomas. As peripheral schwannomas do not usually recur when treated by gross total resection, the use of radiotherapy is not recommended. Conversely, radiotherapy may play a crucial role in VS. Different non-randomized studies have shown the superiority of SRS to microsurgery for patients with VS < 3 cm in preserving short-term facial nerve and hearing function [148,149,150,151]. For large VS, tumor resection followed by SRS or observation are both considered valid options and depend on the size of the residual tumors and neurological symptoms, as well as patient’s preference [141]. The probability to preserve hearing is >75–100% after 2 years, >50–75% after 5 years, and >25–50% after 10 years. After 5 and 10 years, the rates of hearing preservation are similar to patients having microsurgery [152]. The recommendation is to use SRS with a dose of 11–14 Gy at the margin and 11–12 Gy when the risk of hearing loss is a critical issue [141].

Neurofibroma/Perineuroma/Hybrid nerve sheath tumors. Although these tumors comprise rare and different cohorts of benign tumors and prospective evidence is missing, some cautious conclusions can be drawn from the experience of treating vestibular schwannomas. Firstly, if tumors can be easily removed, patients should be advised to undergo surgery. If this is not possible and the patient is symptomatic or symptomatic progression with significant impact on quality of life is expected, radiotherapy should be explored. In small tumors less than 15 mm in size, single-fraction radiosurgery with 12–14 Gy is recommended, while conventionally fractionated stereotactic radiotherapy with 1.8 Gy to 50.4 Gy is applied in larger tumors, especially in areas near critical organs at risk. Recently, hypofractionated stereotactic radiotherapy to reduce overall treatment duration has been explored, e.g., with 5 *×* 5 Gy or 3 *×* 6 Gy, with similar results. With the published retrospective data, 5-year local control of 95% can be expected with the above-mentioned concepts [153].

Neuroendocrine tumors. These benign tumors are most observed in the head and neck region, and the majority of the literature is retrospective and reports good local control after fractionated radiotherapy doses between 40 to 50 Gy with current recommendation of 1.8 Gy to 45 Gy [154]. A case series with varying anatomical site, including retroperitoneal and spinal locations, reported treatment results of 41 patients collected from 1973 to 2015 (highlighting the rarity of such tumors). A 5-year local control of 81% was reported for radiation doses above 53 Gy EQD2Gy compared to 62% for lower radiation doses. Clinical improvement was observed in 94% of symptomatic patients. Thus, if not operable, these tumors can be successfully irradiated with a radiation dose selection depending on the treatment intent (curative vs. palliative symptomatic).

## 9. Medical Treatments

Systemic treatments play a crucial role in VS, PNs, or MPNST, especially when complete resection is not feasible due to the extensive growth and infiltration of surrounding soft tissues.

### 9.1. Vestibular Schwannomas

Bevacizumab is the sole compound that has been successfully used for patients with progressive VS associated with NF2 with remarkable improvement of hearing and objective radiographic responses [155]. Blakeley et al. have conducted a multi-institutional uncontrolled phase 2 study using 7.5 mg/kg bevacizumab administered every 3 weeks in NF2 patients with progressive VS, displaying a hearing improvement in 36% of patients, and a partial radiographic response with volume reduction of 20% or more in 43% (6/14 patients), making bevacizumab a potential treatment option for NF2 patients [156].

### 9.2. Plexiform Neurofibromas

The discovery of the molecular pathogenesis of PN of neurofibromatosis has launched several clinical trials that have investigated targeted therapies. Some initial studies with imatinib (RTK inhibitor of the downstream pathways including MAPK, PI3K/AKT, and JAK/STAT) [157], tipifarnib (RAS inhibitor) [158], pirfenidone (TGF-β inhibitor) [159], sirolimus (mTOR inhibitor) [160,161], interferon alfa-2b inhibitor [162], and everolimus [163] reported limited benefits. Conversely, the development of MEK inhibitors represents the first effective targeted therapy for PN [164]. In this regard, selumetinib is an oral, highly potent, and selective MEK1 inhibitor, that has been investigated primarily in children with remarkable results in terms of disease control and quality of life. In a phase 1 trial on children and adolescents (3–18 years) with NF1 and inoperable PNs, the objective response rate (ORR) was of 71% (15/17 partial response -PR-, and 2 stable disease -SD-) with a median reduction in tumor volume of 31%. Notably, all PR were durable and maintained for a median time of 23 cycles (range 6–42) [165]. The phase 2 trial (SPRINT) on 50 patients aged 2 to 18 with NF1 and inoperable PNs has confirmed the remarkable activity of selumetinib (ORR 68%, median reduction in tumor volume of 27%, median progression-free survival (PFS) not reached at the time of interim-analysis) in association with a clinically meaningful improvement in child-reported interference of pain in daily functioning and overall health-related QoL (38% and 48%, respectively) [166]. Clinical benefit and radiological response with reduction of spinal canal distortion and/or spinal cord deformity was also seen in 18/24 patients with spinal PNs. Furthermore, when selumetinib was employed in asymptomatic PNs, 72% of patients achieved a tumor shrinkage following a median number of 41 cycles (2–67) without additional morbidity or new PN-related symptoms [167]. Building on these results, selumetinib was approved by the Food and Drug Administration (FDA) and European Medicines Agency (EMA) for children ≥ 2 years with NF1 and inoperable and symptomatic PNs in June 2020 and June 2021, respectively [168]. Selumetinib has also been investigated in adults with NF1 with unresectable, symptomatic, and progressive PNs, displaying encouraging activity in a phase 2 trial (ORR 69%; 22% tumor volume shrinkage as best response) with decreased pain intensity [169]. In addition, similar results have been reported in a single-institution experience [170]. Toxicity is mostly mild and reversible in children, including asymptomatic increase in creatine kinase, acneiform or maculopapular rash, paronychia, and gastrointestinal toxicity (diarrhea, nausea, vomiting), while in adults increased liver enzymes in five patients, rash in one patient, and pancreatic enzyme elevation in one patient have been reported.

Mirdametinib is an oral, highly selective small-molecule inhibitor of MEK1 and 2, that has been investigated in 19 patients aged ≥ 16 years with NF1 and progressive and/or symptomatic PNs, showing 2% of PR only, a median reduction in tumor volume of 17.1%, and an improvement of patient-reported outcomes over treatment. The most frequent AEs were acneiform rash, fatigue, and nausea [171]. A further evolution of MEK1-2 inhibitors is represented by trametinib and binimetinib. The interim analysis of the phase 1–2 trial on trametinib in pediatric patients with NF-1–associated PN reported a PR in 12 out of 26 patients (46%) with long-lasting radiological response more than 12 months [172] and manageable side effects, such as paronychia and rash. A recent meta-analysis on efficacy and safety of trametinib from eight studies involving 92 patients with NF-1-associated PNs or low-grade gliomas showed an ORR of 45.3%, a disease control rate of 99.8% with significant activity to stabilize tumor progression, but limited ability to shrink tumors [173]. A higher efficacy has been demonstrated by binimetinib, which yielded a PR in 14 out of 19 patients (73.7%) aged 1–17 years with NF1 and PNs, either progressive or causing significant neurological symptoms; however, a significant burden of grade 3 adverse events was observed, requiring a dose reduction in 13 patients and discontinuation of treatment in 2 other patients [174].

Cabozantinib is an oral multikinase inhibitor with activity against vascular endothelial growth factor (VEGFR) 1 and 2, MET, RET, KIT, AXL, and FLT3. A phase 2 trial (NF-105) in patients aged ≥ 16 years with NF1 and progressive or symptomatic inoperable PNs has been conducted, showing an ORR of 42%, a tumor volume reduction of 15% (from 2.8% to −38.0%), but some concerns in terms of tolerability with 38% of patients who experienced grade 3 adverse events, including palmar-plantar erythrodysesthesia, hypertension, diarrhea, nausea, hypothyroidism, and fatigue, needing a reduction of the daily dose or discontinuation of the treatment [175]. Moreover, no significant improvement in quality of life has been reported; however, an early decrease of intensity of pain (after four cycles) has been observed in patients who achieved a PR [176].

Overall, most of MEK inhibitors and cabozantinib have displayed a remarkable efficacy in decreasing the tumor volume of PNs (ORR ranging from 40% to 74%) with long-lasting tumor response, and reduction of pain intensity without deterioration of QoL. Since radiological response tends to occur later after >1 year of treatment, crucial points are to find biomarkers of response to avoid toxicities in pediatric and adult patients, and also the identification of the optimal timing to discontinue the treatment. Last, considering the activity of these drugs, an open issue is whether there could be a role in a neoadjuvant setting to improve tumor resection. In this regard, some anecdotal reports suggest that MEK inhibitors could have a role, but the tumor shrinkage seems to be limited (15–30%) [177].

### 9.3. Malignant Nerve Sheath Tumors

Given the high risk to progress locally and/or with systemic metastases, adjuvant treatments should be considered in patients who underwent surgery for MPNST regardless of the extent of resection. However, there is still controversy regarding which treatment modality (radiotherapy vs. systemic chemotherapy) and timing (neoadjuvant vs. adjuvant setting) is more effective, due to the lack of definitive data regarding either local disease control or decrease of risk of systemic progression [26]. The key point for the treatment decision is the determination of recurrence risk and prognosis, which is mainly based on different factors, such as large size, high-grade histology, positive margins following resection, presence of necrosis, association with NF1 or previous radiation therapy [178]. A database analysis conducted by the Surveillance, Epidemiology, and End Results (SEER) has built a nomogram that considers six prognostic factors (age, primary site, histologic subtype of MPNST, stage, surgery, and chemotherapy) to predict the outcome (OS) [179] and help clinicians in therapeutic decisions.

Traditional chemotherapy. In general, anthracycline-based treatment is the first-line therapy for unresectable, locally advanced, or metastatic MPNST. The choice of using single-agent anthracycline (doxorubicin) or combined treatment (doxorubicin/ifosfamide) depends on patient-specific factors (i.e., performance status, comorbidities) since the combination of doxorubicin/ifosfamide increases both radiological responses and toxicity, but not OS as compared with single agent doxorubicin [179]. A pooled analysis of 12 EORTC- Soft Tissue and Bone Sarcoma Group (STBSG) trials suggested a minimal superiority of the doxorubicin/ifosfamide regimen over doxorubicin monotherapy in MPNST with median PFS (17 vs. 16 weeks), but not for median OS (48 vs. 51 weeks) [180]. In case of progression following anthracycline-based therapy, other regimens include the topoisomerase II inhibitor etoposide based on the rationale that the topoisomerase-IIα is overexpressed in MPNST [181]. In fact, the SARC006 trial reported that 5/48 patients with MPNST had tumor shrinkage after two cycles of etoposide/ifosfamide after progression following doxorubicin/ifosfamide [182]. Moreover, a case series showed two patients with MPNST with significant responses to etoposide/carboplatin after refractory disease to doxorubicin/ifosfamide [183]. Further cytotoxic chemotherapy regimens can be considered when anthracycline and etoposide-based therapy fails to control the disease; however, limited data on their efficacy have been reported thus far. For instance, gemcitabine in combination with docetaxel was delivered in two patients with MPNST reporting a PR [184], as well as in Japanese retrospective series showed a stabilization of disease in four out of five patients [185]. Last, other compounds, such as carboplatin, dactinomycin, cisplatin, vincristine, cyclophosphamide, imidazole, and carboxamide, have been employed, alone or in combination, for the treatment of MPNST, with disappointing results [186] (see Table 4 for general treatment recommendation).

Targeted therapy. As MPNST are poorly responsive or refractory to traditional chemotherapy, targeted therapy is an appealing opportunity since the Ras/Raf/MEK/ERK is an overexpressed pathway [24]. In fact, some clinical and radiological responses have been reported after MEK inhibitors in MPNST [187,188]. However, MEK inhibitors as single agents promote resistance to therapy [189] as demonstrated by the phase 2 study on sorafenib (multitarget agent, including MEK pathway), reporting an absence of radiological responses in 15 patients [190]. Hence, the use of MEK inhibitors in combination with other targeted therapies should be more effective. Accordingly, there is one phase 2 clinical trial investigating the combination of MEK inhibitor selumetinib and mTOR inhibitor sirolimus in patients with unresectable or metastatic NF1-associated or sporadic MPNSTs (SARC031; NCT03433183). Importantly, as the co-inhibition of MEK and mTOR confers a synergic toxicity, this trial aims to find the optimal daily dose to achieve an effective response while preventing toxicity. The blockade of mTOR pathway only transiently delays tumor recurrence, and thus mTOR inhibitors should be associated with other drugs, and the combination with antiangiogenic compounds have been proposed [191]. However, a phase 2 trial on bevacizumab in combination with mTOR inhibitor everolimus in 25 patients with refractory sporadic or NF1-associated MPNST did not meet the primary endpoint of response rate of ≥16% using WHO criteria, with only 12% of patients achieving an SD for at least 4 months [192]. The combination of mTOR inhibitor sirolimus with the Hsp90 inhibitor ganetespib has been explored in a phase 1–2 trial on 25 MPNST patients, showing no advantage in ORR [193]. The PDGFR and KIT are other druggable pathways in MPNST. The inhibitor imatinib was evaluated in a phase 2 study of 185 metastatic or recurrent soft tissue tumors, including seven MPNST patients, showing poor activity (median PFS of 1.9 months, with stable disease as the best response in one patient) [194]. Likewise, dasatinib did not show any ORR in 14 MPNST patients [195]. Pexidartinib, a selective c-KIT inhibitor conjugated with colony-stimulating factor 1 receptor, in association with the mTOR inhibitor sirolimus, has been investigated in a phase 1 trial, and reported a long-lasting (>18 weeks) stable disease in three out of six patients with MPNST [196], thus leading to further investigation in an ongoing phase 2 trial (NCT02584647). Based on the significant VEGF and PDGFR-alpha protein expression in MPNST, the multi-kinase inhibitor pazopanib was evaluated in a phase 2 trial in 12 patients with advanced MPNST, with a clinical benefit rate of 50% at 12 weeks, a PFS of 5.4 months, and an OS of 10.6 months [197]. Some biological markers, including EGFR, Aurora kinase A (AURKA), or exportin-1 (XPO1), have been demonstrated to be overexpressed in MPNST, thus representing potential druggable pathways. However, the EGFR inhibitor erlotinib [198] and AURKA inhibitor alisertib [199] yielded negative results (PFS 24 and 13 weeks, respectively; OS 48 and 69 weeks, respectively). Conversely, the exportin-1 inhibitor selixenor in combination with doxorubicin achieved 3 PR and 4 SD in nine patients with unresectable or metastatic MPNST, while one patient with NF1-associated MPNST obtained a disease stabilization after selinexor alone, lasting 13.5 months [200]. Neurotropic tropomyosin receptor kinase (NTRK) gene rearrangements have been reported in limited cases of soft-tissue sarcomas. To date, there has been one report only of a dramatic response following entrectinib, lasting 10 months in a heavily pretreated patient with MPNST harboring a novel SNRNP70-NTRK3 fusion gene [201]. Although of significant interest, testing of such a rare molecular alteration in a rare tumor cannot be recommended in daily clinical practice. Since the microenvironment may present an increase of infiltrating cytotoxic T cells and reduction of regulatory T cells, immune-checkpoint inhibitors have been investigated in MPNST. One patient with NF1-associated MPNST received the anti-PD-1 nivolumab based on the detection of an amplification of the chromosomal region 9p23-p24, including the CD274/PD-L1 locus, and obtained a prolonged PR [202]. Similarly, one patient with sporadic metastatic MPNST with positive immunohistochemistry staining for PD-L1 reported a complete response following four cycles of pembrolizumab [203]. To date, two clinical trials are evaluating pembrolizumab alone and neoadjuvant nivolumab plus ipilimumab, respectively, in MPNST (NCT02691026, NCT04465643). Additionally, oncolytic virus therapy is a field of research in unresectable or metastatic MPNST; in this regard, a phase 1 study is recruiting patients for intratumoral administration of an Edmonston strain measles virus genetically engineered to express NF1 (NCT02700230). Other targeted therapeutic strategies under consideration for MPNST include targeting the Wnt/B-catenin and HIPPO signaling pathways, as well as inhibition of BRD4, HDAC, and cyclin-dependent kinase 2 (Table 5).

## 10. Conclusions

Surgery is the therapy of first choice in central and peripheral nerve sheath tumors to obtain a histological diagnosis and reduce tumor burden with the primary aim to preserve surrounding soft tissues and nerve functioning. In this regard, gross total resection may be curative for benign tumors. Additionally, the extent of resection is crucial for reducing residual tumors and is correlated with OS in MPNST. Given the heterogeneity and rarity of these tumors, there is a paucity of well-powered clinical trials, thus it is not possible to generate evidence-based treatment recommendations for non-surgical modalities. However, some clinical trials have been reported on targeted therapies in plexiform neurofibromas of NF1 patients or in heterogenous cohorts of soft-tissue tumors, including MPNSTs, with initial data of efficacy that need to be further investigated.

## Figures and Tables

**Table 1:** Epidemiology and clinical features.

Tumor Type	Estimated Incidence	Age	Sex	Location	Clinical Presentation
Schwannoma	1.09 per 100,000/year	All age groups are affected, with a peak incidence in the 4th–6th decades	No sex predilection	- Skin and subcutaneous tissues of the head and neck, or along the flexor surfaces of the extremities - Spinal intradural extramedullary site with growth into foraminal space - Eight cranial nerve (bilateral involvement in NF2)	- Asymptomatic lesion - Radicular pain, sensory or motor symptoms - Unilateral sensorineural hearing loss, tinnitus, vertigo, unsteadiness in vestibular schwannoma. Trigeminal and facial neuropathies, brainstem compression and hydrocephalus in large lesion.
Neurofibroma (Localized, diffuse, plexiform subtype)	5.3% of all benign soft tissue tumors	All age groups are equally affected	No sex predilection	- Skin, with predominant dermal involvement, less frequently medium-sized nerves, a nerve plexus, a major nerve trunk, or spinal nerve roots - Bilateral and/or multiple spinal root involvement in NF1 - Spinal cord compression - Cranial nerve involvement is ultrarare	- Asymptomatic, soft, mobile lesion - Sensory or motor symptoms when trunk nerve is affected - Pain - Urinary or bowel obstruction with sensory or motor symptoms - Craniofacial disfigurement with impairment of function (visual impairment, upper airway compression)
Perineurioma (Intraneural and soft tissue subtypes)	1% of nerve sheath and soft tissue neoplasms, respectively (>50 cases of intraneural perineuriomas and >300 cases of soft tissue perineuriomas have been described)	- Intraneural perineurioma: young adults and adolescents; rare in children - Soft tissue perineurioma: peak in middle-aged adults	Slight prevalence for female (M:F ratio 1:2)	- Common presentation: focal, unilateral lesion affecting major peripheral nerves (sciatic, median, radial, brachial plexus) and their branches. - Uncommon locations: cranial nerves, lateral ventricle, oral cavity, skin, and mandible. Bilateral or unilateral multifocal lesions are rare	- Painless mononeuropathy with muscle atrophy and fusiform nerve enlargement - Sensory symptoms or pain are rare. - Limb undergrowth or hand/foot discrepancy in in 25% of patients with proximal lesions of lower extremities
Hybrid nerve sheath tumor	Very rare	Over a wide age range, with a peak in young adults	Equal sex distribution	- The most common site is the fingers - Rare cases of cranial nerves involvement	- Painless mass in most of cases - When large peripheral nerves or spinal nerves are involved, the tumor may be associated with pain or neurological impairment
Malignant peripheral nerve sheath tumour (MPNST) (epithelioid and perineural subtypes)	2–10% of soft tissue sarcomas. Epithelioid MPNST is particularly rare (~5% of all MPNST)	Sporadic MPNST occurs most commonly in patients aged 20–50 years. MPNSTs in children are usually associated with NF1	Similar sex distribution (M:F 1.2:1)	- Extremities, trunk, head, and neck area	- Large mass involving surrounding soft tissue - Pain or neuropathic symptoms
Malignant melanotic nerve sheath tumour (MMNST)	Very rare	Median age of 22.5 years in patients with Carney complex and 33.2 years in patients with sporadic presentation	No sex predilection	- Common sites are spinal or autonomic nerves near the midline - Uncommon sites: gastrointestinal tract, bone, soft tissues, heart, bronchus, liver, and skin	- Mass effect pain, sensory symptoms, bone erosion - Respiratory and liver failure have been reported in metastatic disease
Neuroendocrine tumour (previously paraganglioma)	Very rare	40–60 years	No sex predilection	- Cauda equina region	- Low back pain and sciatica - Less frequent: spinal cord symptoms or cauda equina syndrome - Rare: papilledema and increased intracranial pressure

**Table 2:** Recommendations for pathological diagnosis according to WHO 2021.

Tumor Type	Malignancy *	Essential Diagnostic Criteria
Schwannoma	Benign	Extensive S100 and SOX10 expression; Verocay bodies; hyalinized blood vessels; loss of INI1 expression (epithelioid schwannoma) of mosaic pattern of INI1 expression (syndrome-associated schwannoma)
Neurofibroma	Benign	Infiltrative, low-cellularity spindle cell neoplasm associated with a variably myxoid to collagenous stroma and a mixed cell population
Perineurioma	Benign	Slender spindle cells with bipolar cytoplasmic processes in a storiform and/or whorled architecture or pseudo-onion bulb pattern positive for at least one perineurial antigen and negative for S100
Hybrid nerve sheath tumors	Benign	Intermingled features of two types of benign nerve sheath tumors; appropriate immunohistochemical staining for each component
MPNST	Malignant	For patients with NF1, a histopathological consistent malignant spindle cell tumor is sufficient. For sporadic tumors, additional focal S100 or SOX10 expression and association with a peripheral nerve and no (X;18)(p11.2;q11.2) translocation (oncogenic SS18-SSX1 fusion) of association with a peripheral nerve and immunohistochemical/molecular proof of PRC2 inactivation or methylation profile of MPNST
Epithelioid MPNST	Malignant	Epithelioid cells with prominent nucleoli showing diffuse expression of S100, absence of melanocytic markers, and SMARCB1 loss
Malignant melanotic nerve sheath tumor	Malignant	Fascicular to sheet-like proliferation of variably pigmented, relatively uniform plump spindled cells with coexpression of S100/SOX10 and melanocytic markers or PRKAR1A mutation Desirable: origin from paraspinal or visceral autonomic nerves
Cauda equina neuroendocrine tumor	Benign	Well-demarcated tumor of the cauda equina with a nested pattern and synaptophysin or chromogranin expression

* Formally, for most of these tumors, there is no overall CNS WHO grade as neoplasms should be graded preferably within each tumor type.

**Table 3:** Impact of surgery on outcome (gross total vs. partial resection/inoperable).

Tumor Type	Impact of Gross Total Resection	Consequences of Residual Tumor	Indications for Radiotherapy
Schwannoma	- Potential cure with long-term tumor control - Pain relief	- Unpleasant cosmetic appearance	- Commonly not indicated
Vestibular schwannoma(VS)	- Potential cure with long-term tumor control - Tumor debulking in case of large lesion occupying the cerebellopontine cistern, with or without brainstem and cranial nerves displacement - Large tumor with brainstem and cranial nerve displacement	- Persisting hearing loss and/or facial palsy - Local tumor regrowth	- For smaller VS where preserving facial nerve and hearing function is the primary goal of treatment - For large VS as adjuvant treatment following surgery
Neurofibroma	- Potential cure with long-term tumor control - Pain relief	- Unpleasant cosmetic appearance - Local tumor progression - Malignant transformation in case of plexiform neurofibroma, ANNUBP	- Commonly not indicated
Perineurioma	- Potential cure with long-term tumor control - Pain reduction	- Unpleasant cosmetic appearance - Local tumor regrowth	- Commonly not indicated
Hybrid nerve sheath tumors	- Potential cure with long-term tumor control - Pain reduction	- Unpleasant cosmetic appearance - Local tumor regrowth	- Commonly not indicated
MPNST	- Benefit over subtotal resection in local disease control and overall survival	- -Local tumor progression - Systemic metastases	- Any case of MPNST, even if resected completed. Inoperable or recurrent tumors
Cauda equina neuroendocrine tumor	- Potential cure with long-term tumor control - Pain relief and/or reduction of cauda equine syndrome	- Local tumor progression - CSF spread	- Incompletely resected tumors - Inoperable or recurrent tumors

**Table 4:** General treatment recommendations.

	Class of Evidence	Level of Recommendation
Resection is recommended to obtain a histological and molecular diagnosis.	II	B
Gross total resection is recommended as therapy of first choice when feasible in PNST. When risk of neurological sequelae from surgery is high, detailed informed preoperative counseling by a surgeon experienced in performing such surgery is important.	III	B
Use of intraoperative electrophysiological monitoring is mandatory to preserve nerve functioning during surgery of PNST.	III	B
Intraoperative high-resolution ultrasound and/or use of a microscope are recommended to achieve complete resection by intracapsular dissection and preserving the attached functional nerve fibers.	IV	Good practice point
Observaton with serial MRI and audiological monitoring without any tumor-directed treatment is considered appropriate for incidental, asymptomatic vestibular schwannomas	III	C
Surgery is considered the primary treatment to reduce mass effect in vestibular schwannomas	II	B
To improve the rate of functional preservation, intraoperative monitoring is mandatory for surgery of vestibular schwannomas and should include somatosensoric evoked potentials, monitoring of the facial nerve comprising direct electrical stimulation and free-running electromyography, and brainstem auditory evoked responses	III	B
The frequency of surveillance imaging with MRI should be based on the extent of resection (GTR vs. non-GTR) and tumor aggressiveness, and the duration should be up to 5 years in PNST	IV	Good practice point
Annual MRI is recommended for 5 years in patients with untreated, incidental schwannomas, as well as conservatively treated, irradiated, and incompletely resected vestibular schwannomas. Thereafter, the follow-up intervals can be increased	IV	Good practice point
Repeated surgery in patients with local tumor progression or recurrence of PNST should be considered	IV	Good practice point
Radiotherapy may be omitted in benign tumors after complete resection (e.g., schwannomas)	II	B
SRS is superior over microsurgery for patients with vestibular schwannomas < 3 cm in terms of preserving facial nerve and hearing function	II	B
SRS should be delivered with a dose of 11–14 Gy at the margin in vestibular schwannomas and 11–12 Gy when the risk of hearing loss is a critical issue	III	C
Radiotherapy is not recommended for NF-related plexiform neurofibromas given the theoretic risk of secondary malignancy in a tumor-suppressor syndrome	IV	Good practice point
Perineurioma/hybrid nerve sheath tumors with high risk of neurological sequelae after surgery less than 15 mm in size may be treated with a single fraction radiosurgery with 12–14 Gy, while fractionated stereotactic radiotherapy with 1.8 Gy to 50.4 Gy may be considered for larger tumors	IV	C
Radiotherapy (55.8 Gy in 1.8 Gy) should be considered in MPNST after incomplete resection or greater than 5 cm, and pre-operatively in case of unresectable MPNST to improve local control and/or increase the potential of an R1 or even R0 resection	IV	C
Final dose of adjuvant radiotherapy after surgery of MPNST may be determined by the resection status: a boost of 10.8 Gy (R1 resection; cumulative dose 55.8 Gy) or 19.8 Gy (R2 resection; cumulative dose 64.8 Gy) should be considered if necessary	IV	C
If not operable, cauda equine neuroendocrine tumors may be treated with a radiation dose depending on the treatment intent (curative vs. palliative symptomatic)	IV	C
Consider bevacizumab in patients with multiple rapidly enlarging tumours, who are inoperable (e.g., bilateral vestibular schwannomas)	II	B
In patients with plexiform neurofibromas or MPNSTs, systemic treatments should be considered especially when complete resection is not feasible	II	B
Consider targeted therapy with MEK inhibitor selumetinib in children ≥ 2 years with NF1 and inoperable and symptomatic plexiform neurofibromas	I	B
Consider targeted therapy with MEK inhibitor selumetinib NF1 adult patients with unresectable, symptomatic, and/or progressive plexiform neurofibromas	II	B
Anthracycline-based treatment is the first-line therapy for unresectable, locally advanced, or metastatic MPNST	II	B
The topoisomerase II inhibitor etoposide, alone or in association with ifosfamide, could be considered in case of progression of MPNST following anthracycline-based therapy	III	C
Other cytotoxic chemotherapy regimens, including gemcitabine plus docetaxel, may be considered when anthracycline and etoposide-based therapy fails to control MPNST	IV	C
Sequencing to identify molecular targets to direct potential targeted therapy may be performed for incompletely resected or recurrent tumors that have exhausted treatment options	III	C
In patients with recurrent plexiform neurofibromas or MPNST who are no longer eligible for local treatments, enrollment in clinical trials might be warranted, particularly in patients with a good performance status.	II	C

**Table 5:** Medical treatments: ongoing clinical trials.

Study	Phase	N° of Patients	Treatment	Outcome Measure
Cutaneous neurofibroma				
NCT04730583	1	20	- Kybella injection - 980 nm pulse laser - 755 nm Alexandrite Laser - Radiofrequency	Primary: - incidence of treatment-emergent adverse events Secondary: - Patient-reported outcomes - Clinician-reported outcomes
NCT05199376 CryoNF1	NA	30	- Percutaneous cryotherapy	Primary: - improvement in physical health-related quality of life Secondary: - tumor response (REiNS and RECIST 1.1 criteria) - functional discomfort - patients’ pain - safety - patients’ satisfaction and self-esteem - need for multiple percutaneous cryotherapy procedures
NCT05005845	2	168	- 0.5% NFX-179 gel - 1.5% NFX-179 gel - Drug: Vehicle gel	Primary: - Safety Secondary: - Number of patients with at least 50% of tumor volume reduction - Percentage of reduction of tumor volume
NCT02728388	2	30	- Photodynamic therapy plus aminolevulinic acid - Photodynamic therapy plus topical gel (placebo)	Primary: - Time to disease progression Secondary: - Tumor growth rate
Atypical/plexiform neurofibroma				
NCT04750928	1/2	50	- Abemaciclib	Primary: - Safety Secondary: - Response rate - Measurement of phosphorylated Retinoblastoma in tumor biopsy samples to measure the effect of CDK4/6 target inhibition - Drug level in blood - Pain and quality of life
NCT04954001	1/2	160	- FCN-159	Primary: - MTD - DLT - RP2D - ORR Secondary: - Safety - Changes in neurinoma-related symptoms - Patient- and observer-reported outcomes and functional measures
NCT05309668 (SPRINKLE)	1/2	44	- Selumetinib granule formulation	Primary: - AUC - Safety Secondary: - Self-reported palatability - N-desmethyl selumetinib AUC derived after single- and multiple-dose administrations to derive PK of the granule formulation
NCT03326388 (INSPECT)	1/2	30	- Intermitting dose of selumetinib	Primary: - MTD Secondary: - ORR - Cardiac function (fractional shortening, QTc) - Retinal detachment - Other adverse events - Cmax - Tmax - AUC - Time to progression - Pain evaluation - Evaluation of effect on disfigurement - Quality of life
NCT02390752	1/2	81	- PLX 3397	Primary: - ORR - Safety Secondary: - PK profile - effect of PLX 3397 on circulating biomarkers
NCT03363217	2	150 (including also LGG and HGG MAPK/ERK mutated)	- Trametinib	Primary: - ORR Secondary: - Time to progression - PFS - OS - Safety - Serum level of trametinib - Quality of life evaluation
Malignant peripheral nerve sheath tumor				
NCT02584647	1/2	43	- PLX3397 plus sirolimus	Primary: - MTD - PFS Secondary: - OS
NCT05107037	1	120	- TQ-B3234 capsule	Primary: - MTD - DLT - RP2D - ORR Secondary: - Cmax - Tmax - t1/2 - AUC - DF - safety
NCT05011019 (Chinese patients)	1/2	192	- AL2846 capsules	Primary: - Safety - ORR Secondary: - RP2D - PFS - DOR - OS - Pain Scale (self-report form) - Quality of life
NCT02700230	1	30	- Intratumoral MV-NIS (vaccine therapy)	Primary: - Best response using the WHO response criteria - Safety - MTD Secondary: - Quality of life - Change in biodistribution of virally infected cells at various time points after infection with MV-NIS - Growth rate between treated and untreated lesions - Humoral and cellular immune response to the injected virus - Incidence of viremia, measles virus shedding/persistence, or replication following intratumoral administration - PFS - Time to progression
NCT05245500	1/2	339 (including also solid tumors with MTAP deletion)	- MRTX1719	Primary: - Safety - DLT - ORR - DOR - PFS - OS Secondary: - Cmax - Tmax - t1/2 - AUC - plasma clearance and volume distribution after oral administration of drug
NCT04917042	2	24	- Tazemetostat	Primary: - ORR Secondary: - PFS - Time to progression - Clinical benefit
NCT04897321	1	32 (including also B7-H3+ solid tumors)	- B7-H3 CAR T cells - Fludarabine - Cyclophosphamide - MESNA	Primary: - MTD Secondary: - Clinical response (RECIST criteria)
NCT04872543	2	25	- STX727 plus pegfilgrastim	Primary: - Best clinical benefit Secondary: - ORR
NCT04465643	2	18	- Neoadjuvant Nivolumab plus ipilimumab	Primary: - Safety - MTD Secondary: - number of treatment-emergent adverse events - ORR - Change in pain levels - PFS - Tumor response as assessed by immune markers in tumor samples - Pharmacodynamic activity as assessed by markers in blood samples
NCT04420975	1	20 (including also other soft tissue tumors)	- Nivolumab and BO-112 before surgery	Primary: - Incidence of adverse events Secondary: - Immune-oncologic impact of the BO-112 alone or in combination with nivolumab
NCT04222413	1	54 (including also other advanced or metastatic solid tumors)	- Metarrestin (ML-246)	Primary: - MTD - ORR Secondary: - PK - RP2D - DOR - PFS
NCT03618381	1	36 (including also other soft tissue tumors)	- EGFR806 CAR T cell	Primary: - MTD - DLT Secondary: - persistence of CAR T cells in the peripheral blood and in blood marrow
NCT03611868	1/2	224 (including also metastatic melanoma or advanced solid tumors)	- APG 115 plus pembrolizumab	Primary: - MTD - RP2D - ORR Secondary: - Not reported

NA: not applicable; MTD: maximum tolerated dose; DLT: dose-limiting toxicity; RP2D: phase 2 recommended clinical dose; ORR: objective response rate; Cmax: peak concentration; Tmax: time to maximum plasma concentration; t1/2: clearance half-life; AUC: area under plasma concentration-time curve; DF: coefficient of fluctuation; PFS: progression-free survival; OS: overall survival; DOR: duration of response; PK: pharmacokinetic; WHO: World Health Organization.

## Data Availability

No new data were created or analyzed in this study. Data sharing is not applicable to this article.

## References

[1] Louis D.N., Perry A., Wesseling P., Brat D.J., Cree I.A., Figarella-Branger D., Hawkins C., Ng H.K., Pfister S.M., Reifenberger G. (2021). The 2021 WHO Classification of Tumors of the Central Nervous System: A summary. Neuro-Oncology.

[2] The WHO Classification of Tumours Editorial Board (2020). WHO Classification of Tumours Soft Tissue and Bone Tumours.

[3] Ostrom Q.T., Cioffi G., Waite K., Kruchko C., Barnholtz-Sloan J.S. (2021). CBTRUS Statistical Report: Primary Brain and Other Central Nervous System Tumors Diagnosed in the United States in 2014–2018. Neuro-Oncology.

[4] Brainin M., Barnes M., Baron J.C., Gilhus N.E., Hughes R., Selmaj K., Waldemar G., Guideline Standards Subcommittee of the EFNS Scientific Committee (2004). Guidance for the preparation of neurological management guidelines by EFNS scientific task forces—Revised recommendations 2004. Eur. J. Neurol..

[5] Casadei G.P., Komori T., Scheithauer B.W., Miller G.M., Parisi J.E., Kelly P.J. (1993). Intracranial parenchymal schwannoma. A clinico-pathological and neuroimaging study of nine cases. J. Neurosurg..

[6] Voltaggio L., Murray R., Lasota J., Miettinen M. (2012). Gastric schwannoma: A clinicopathologic study of 51 cases and critical review of the literature. Hum. Pathol..

[7] Babu R., Sharma R., Bagley J.H., Hatef J., Friedman A.H., Adamson C. (2013). Vestibular schwannomas in the modern era: Epidemiology, treatment trends, and disparities in management. J. Neurosurg..

[8] Evans D.G.R. (2009). Neurofibromatosis type 2 (NF2): A clinical and molecular review. Orphanet J. Rare Dis..

[9] Andersen J.F., Nilsen K.S., Vassbotn F.S., Møller P., Myrseth E., Lund-Johansen M., Goplen F.K. (2015). Predictors of Vertigo in Patients with Untreated Vestibular Schwannoma. Otol. Neurotol..

[10] Woodruff J.M., Selig A.M., Crowley K., Allen P.W. (1994). Schwannoma (Neurilemoma) with Malignant Transformation A Rare, Distinctive Peripheral Nerve Tumor. Am. J. Surg. Pathol..

[11] McMenamin M.E., Fletcher C.D.M. (2001). Expanding the Spectrum of Malignant Change in Schwannomas: Epithelioid malignant change, epithelioid malignant peripheral nerve sheath tumor, and epithelioid angiosarcoma: A study of 17 cases. Am. J. Surg. Pathol..

[12] Carter J.M., O’Hara C., Dundas G., Gilchrist D., Collins M.S., Eaton K., Judkins A.R., Biegel J.A., Folpe A.L. (2012). Epithelioid Malignant Peripheral Nerve Sheath Tumor Arising in a Schwannoma, in a Patient With “Neuroblastoma-like” Schwannomatosis and a Novel Germline SMARCB1 Mutation. Am. J. Surg. Pathol..

[13] Perry A., Reuss D.E., Rodriguez F. (2020). Neurofibroma. WHO Classification of Tumours Series.

[14] Wolkenstein P., Zeller J., Revuz J., Ecosse E., Leplège A. (2001). Quality-of-Life Impairment in Neurofibromatosis Type 1: A cross-sectional study of 128 cases. Arch. Dermatol..

[15] Meyer A., Billings S.D. (2020). What’s new in nerve sheath tumors. Virchows Arch..

[16] Collins-Sawaragi Y.C., Ferner R., Vassallo G., De Agrò G., Eccles S., Cadwgan J., Hargrave D., Hupton E., Eelloo J., Lunt L. (2022). Location, symptoms, and management of plexiform neurofibromas in 127 children with neurofibromatosis 1, attending the National Complex Neurofibromatosis 1 service, 2018–2019. Am. J. Med. Genet. Part A.

[17] Miettinen M.M., Antonescu C.R., Fletcher C.D., Kim A., Lazar A.J., Quezado M.M., Reilly K.M., Stemmer-Rachamimov A., Stewart D.R., Viskochil D. (2017). Histopathologic evaluation of atypical neurofibromatous tumors and their transformation into malignant peripheral nerve sheath tumor in patients with neurofibromatosis 1—A consensus overview. Hum. Pathol..

[18] Stewart D.R., Korf B.R., Nathanson K.L., Stevenson D.A., Yohay K. (2018). Care of adults with neurofibromatosis type 1: A clinical practice resource of the American College of Medical Genetics and Genomics (ACMG). Anesth. Analg..

[19] Bs J.E.B., Peterson C.R., Dhakal S., Giampoli E.J., Constine L.S. (2014). Malignant peripheral nerve sheath tumors (MPNST): A SEER analysis of incidence across the age spectrum and therapeutic interventions in the pediatric population. Pediatr. Blood Cancer.

[20] Somatilaka B.N., Sadek A., McKay R.M., Le L.Q. (2022). Malignant peripheral nerve sheath tumor: Models, biology, and translation. Oncogene.

[21] Ferner R.E., Gutmann D.H. (2002). International consensus statement on malignant peripheral nerve sheath tumors in neurofibroma-tosis. Cancer Res..

[22] Evans D.G.R., Baser M.E., McGaughran J., Sharif S., Howard E., Moran A. (2002). Malignant peripheral nerve sheath tumours in neurofibromatosis 1. J. Med. Genet..

[23] Foley K.M., Woodruff J.M., Ellis F.T., Posner J.B. (1980). Radiation-induced malignant and atypical peripheral nerve sheath tumors. Ann. Neurol..

[24] Carli M., Ferrari A., Mattke A., Zanetti I., Casanova M., Bisogno G., Cecchetto G., Alaggio R., De Sio L., Koscielniak E. (2005). Pediatric Malignant Peripheral Nerve Sheath Tumor: The Italian and German Soft Tissue Sarcoma Cooperative Group. J. Clin. Oncol..

[25] Wong W.W., Hirose T., Scheithauer B.W., Schild S., Gunderson L.L. (1998). Malignant peripheral nerve sheath tumor: Analysis of treatment outcome. Int. J. Radiat. Oncol..

[26] Stucky C.-C.H., Johnson K.N., Gray R.J., Pockaj B.A., Ocal I.T., Rose P.S., Wasif N. (2011). Malignant Peripheral Nerve Sheath Tumors (MPNST): The Mayo Clinic Experience. Ann. Surg. Oncol..

[27] Valentin T., Le Cesne A., Ray-Coquard I., Italiano A., Decanter G., Bompas E., Isambert N., Thariat J., Linassier C., Bertucci F. (2016). Management and prognosis of malignant peripheral nerve sheath tumors: The experience of the French Sarcoma Group (GSF-GETO). Eur. J. Cancer.

[28] Torres-Mora J., Dry S., Li X., Binder S., Amin M., Folpe A.L. (2014). Malignant Melanotic Schwannian Tumor: A clinicopathologic, im-munohistochemical, and gene expression profiling study of 40 cases, with a proposal for the reclassification of “melanotic schwannoma”. Am. J. Surg. Pathol..

[29] Carney J.A. (1990). Psammomatous melanotic schwannoma. A distinctive, heritable tumor with special associations, including cardiac myxoma and the Cushing syndrome. Am. J. Surg. Pathol..

[30] Zhang H.-Y., Yang G.-H., Chen H.-J., Wei B., Ke Q., Guo H., Ye L., Bu H., Yang K., Zhang Y.-H. (2005). Clinicopathological, immunohistochemical, and ultrastructural study of 13 cases of melanotic schwannoma. Chin. Med. J..

[31] Wang L., Zehir A., Sadowska J., Zhou N., Rosenblum M., Busam K., Agaram N., Travis W., Arcila M., Dogan S. (2015). Consistent copy number changes and recurrentPRKAR1Amutations distinguish Melanotic Schwannomas from Melanomas: SNP-array and next generation sequencing analysis. Genes Chromosom. Cancer.

[32] Chen Y.-Y., Yen H.-H., Soon M.-S. (2007). Solitary gastric melanotic schwannoma: Sonographic findings. J. Clin. Ultrasound.

[33] Chetty R., Vajpeyi R., Penwick J.L. (2007). Psammomatous melanotic schwannoma presenting as colonic polyps. Virchows Arch..

[34] Lindholm K., Moran C.A. (2018). Primary mediastinal melanotic schwannian tumors: A clinicopathological and immunohistochemical study of 5 cases. Ann. Diagn. Pathol..

[35] Khoo M., Pressney I., Hargunani R., Tirabosco R. (2016). Melanotic schwannoma: An 11-year case series. Skelet. Radiol..

[36] Lenartowicz K.A., Goyal A., Mauermann M.L., Wilson T.J., Spinner R.J. (2021). Clinical Features, Natural History, and Outcomes of Intraneural Perineuriomas: A Systematic Review of the Literature. World Neurosurg..

[37] Almefty R., Webber B.L., Arnautović K.I. (2006). Intraneural perineurioma of the third cranial nerve: Occurrence and identification: Case report. J. Neurosurg..

[38] Christoforidis M., Buhl R., Paulus W., Sepehrnia A. (2007). intraneural perineurioma of the viiith cranial nerve: Case report. Neurosurgery.

[39] Giannini C., Scheithauer B.W., Steinberg J., Cosgrove T.J. (1998). Intraventricular Perineurioma: Case Report. Neurosurgery.

[40] Mauermann M.L., Amrami K.K., Kuntz N.L., Spinner R.J., Dyck P.J., Bosch E.P., Engelstad J., Felmlee J.P., Dyck P.J. (2009). Longitudinal study of intraneural perineurioma--a benign, focal hypertrophic neuropathy of youth. Brain.

[41] Boyanton B.L., Jones J.K., Shenaq S.M., Hicks M.J., Bhattacharjee M.B. (2007). Intraneural Perineurioma: A Systematic Review with Illustrative Cases. Arch. Pathol. Lab. Med..

[42] Wilson T.J., Howe B.M., Stewart S.A., Spinner R.J., Amrami K.K. (2018). Clinicoradiological features of intraneural perineuriomas obviate the need for tissue diagnosis. J. Neurosurg..

[43] Pendleton C., Lenartowicz K.A., Howe B.M., Spinner R.J. (2020). Limb Undergrowth in Intraneural Perineuriomas: An Under-Recognized Association. World Neurosurg..

[44] Schaefer I.-M., Ströbel P., Thiha A., Sohns J.M., Mühlfeld C., Küffer S., Felmerer G., Stepniewski A., Pauli S., Agaimy A. (2013). Soft tissue perineurioma and other unusual tumors in a patient with neurofibromatosis type 1. Int. J. Clin. Exp. Pathol..

[45] Al-Adnani M. (2017). Soft Tissue Perineurioma in a Child with Neurofibromatosis Type 1: A Case Report and Review of the Literature. Pediatr. Dev. Pathol..

[46] White B., Belzberg A., Ahlawat S., Blakeley J., Rodriguez F.J. (2020). Intraneural perineurioma in neurofibromatosis type 2 with molecular analysis. Clin. Neuropathol..

[47] Pendleton C., Spinner R.J., Dyck P.J.B., Mauermann M.L., Ladak A., Restrepo C.E., Baheti S., Klein C.J. (2020). Association of intraneural perineurioma with neurofibromatosis type 2. Acta Neurochir..

[48] Hornick J.L., Bundock E.A., Fletcher C.D.M. (2009). Hybrid Schwannoma/Perineurioma: Clinicopathologic analysis of 42 distinctive benign nerve sheath tumors. Am. J. Surg. Pathol..

[49] Michal M., Kazakov D.V., Belousova I., Bisceglia M., Zamecnik M., Mukensnabl P. (2004). A benign neoplasm with histopathological features of both schwannoma and retiform perineurioma (benign schwannoma-perineurioma): A report of six cases of a distinctive soft tissue tumor with a predilection for the fingers. Virchows Arch..

[50] Goyal-Honavar A., Gupta A., Chacko G., Chacko A.G. (2021). Trigeminal hybrid nerve sheath tumor—A case report and literature review. Br. J. Neurosurg..

[51] Las Heras F.L., Martuza R., Caruso P., Rincon S., Stemmer-Rachamimov A. (2013). 24-Year-Old Woman with An Internal Auditory Canal Mass. Hybrid peripheral nerve sheath tumor with schwannoma/perineurioma components. Brain Pathol..

[52] Matsuo S., Higaki R., Matsukado K. (2021). Microsurgical Resection of Hybrid Nerve Sheath Tumor Involving the Orbit and Lateral Wall of the Cavernous Sinus: 2-Dimensional Operative Video. Oper. Neurosurg..

[53] Harder A., Wesemann M., Hagel C., Schittenhelm J., Fischer S., Tatagiba M., Nagel C., Jeibmann A., Bohring A., Mautner V.-F. (2012). Hybrid Neurofibroma/Schwannoma is Overrepresented Among Schwannomatosis and Neurofibromatosis Patients. Am. J. Surg. Pathol..

[54] MacCollin M., Chiocca E.A., Evans G., Friedman J., Horvitz R., Jaramillo D., Lev M., Mautner V.F., Niimura M., Plotkin S.R. (2005). Diagnostic criteria for schwannomatosis. Neurology.

[55] Kacerovska D., Michal M., Kuroda N., Tanaka A., Sima R., Denisjuk N., Kreuzberg B., Ricarova R., Kazakov D.V. (2013). Hybrid Peripheral Nerve Sheath Tumors, Including a Malignant Variant in Type 1 Neurofibromatosis. Am. J. Dermatopathol..

[56] Inatomi Y., Ito T., Nagae K., Yamada Y., Kiyomatsu M., Nakano-Nakamura M., Uchi H., Oda Y., Furue M. (2014). Hybrid perineurioma-neurofibroma in a patient with neurofibromatosis type 1, clinically mimicking malignant peripheral nerve sheath tumor. Eur. J. Dermatol..

[57] Engelhard H.H., Villano J.L., Porter K.R., Stewart A.K., Barua M., Ii F.G.B., Newton H.B., Takashima H., Takebayashi T., Yoshimoto M. (2010). Clinical presentation, histology, and treatment in 430 patients with primary tumors of the spinal cord, spinal meninges, or cauda equina. J. Neurosurg. Spine.

[58] Palmisciano P., Sagoo N.S., Haider A.S., Ogasawara C., Ogasawara M., Bin Alamer O., Heidari K.S., Raj K.M., Scalia G., Umana G.E. (2022). Primary Paraganglioma of the Spine: A Systematic Review of Clinical Features and Surgical Management in Cauda Equina versus Non–Cauda Equina Lesions. World Neurosurg..

[59] Shtaya A., Iorga R., Hettige S., Bridges L.R., Stapleton S., Johnston F.G. (2021). Paraganglioma of the cauda equina: A tertiary centre experience and scoping review of the current literature. Neurosurg. Rev..

[60] Blades D.A., Hardy R.W., Cohen M. (1991). Cervical paraganglioma with subsequent intracranial and intraspinal metastases. J. Neurosurg..

[61] Sachani H., Tripathi M., Madhusudan K.S., Semalti K., Shanker S., ArunRaj S.T., Bal C. (2021). Thoracic Extradural Paraganglioma Localized on 68Ga-DOTANOC PET/CT. Clin. Nucl. Med..

[62] Gelabert-González M. (2005). Paragangliomas of the lumbar region. Report of two cases and review of the literature. J. Neurosurg. Spine.

[63] Adriani K.S., Stenvers D.J., Imanse J.G. (2012). Pearls & Oy-sters: Lumbar paraganglioma: Can you see it in the eyes?. Neurology.

[64] Moran C., Rush W., Mena H. (1997). Primary spinal paragangliomas: A clinicopathological and immunohistochemical study of 30 cases. Histopathology.

[65] Roche P.H., Figarella-Branger D., Regis J., Peragut J.C. (1996). Cauda equina paraganglioma with subsequent intracranial and intraspinal metastases. Acta Neurochir..

[66] Thines L., Lejeune J.P., Ruchoux M.M., Assaker R. (2006). Management of delayed intracranial and intraspinal metastases of intradural spinal paragangliomas. Acta Neurochir..

[67] Thomson N., Pacak K., Schmidt M.H., Palmer C.A., Salzman K.L., Champine M., Schiffman J.D., Cohen A.L. (2017). Leptomeningeal dissemination of a low-grade lumbar paraganglioma: Case report. J. Neurosurg. Spine.

[68] Sonneland P.R., Scheithauer B.W., LeChago J., Crawford B.G., Onofrio B.M. (1986). Paraganglioma of the cauda equina region. Clinico-pathologic study of 31 cases with special reference to immunocytology and ultrastructure. Cancer.

[69] Easton D.F., Ponder M.A., Huson S.M., Ponder B.A. (1993). An analysis of variation in expression of neurofibromatosis (NF) type 1 (NF1): Evidence for modifying genes. Am. J. Hum. Genet..

[70] Jordan J.T., Plotkin S.R. (2022). Neurofibromatoses. Hematol. Oncol. Clin. N. Am..

[71] Legius E., Messiaen L., Wolkenstein P., Pancza P., Avery R.A., Berman Y., Blakeley J., Babovic-Vuksanovic D., Cunha K.S., Ferner R. (2021). Revised diagnostic criteria for neurofibromatosis type 1 and Legius syndrome: An international consensus recommendation. Anesth. Analg..

[72] Messiaen L., Tadini G., Legius E., Brems H. (2020). Multidiscipilinary Approach to Neurofibromatosis 1. Molecular Diagnosis of NF1.

[73] Carton C., Evans D.G., Blanco I., Friedrich R.E., Ferner R.E., Farschtschi S., Salvador H., Azizi A.A., Mautner V., Röhl C. (2023). ERN GENTURIS tumour surveillance guidelines for individuals with neurofibromatosis type 1. Eclinicalmedicine.

[74] Plotkin S.R., Messiaen L., Legius E., Pancza P., Avery R.A., Blakeley J.O., Babovic-Vuksanovic D., Ferner R., Fisher M.J., Friedman J.M. (2022). Updated diagnostic criteria and nomenclature for neurofibromatosis type 2 and schwannomatosis: An international consensus recommendation. Anesth. Analg..

[75] Burns R., Niendorf K., Steinberg K., Mueller A., Ly I., Jordan J.T., Plotkin S.R., Hicks S.R. (2022). Genetic testing to gain diagnostic clarity in neurofibromatosis type 2 and schwannomatosis. Am. J. Med. Genet. Part A.

[76] Evans D.G., Hartley C.L., Smith P.T., King A.T., Bowers N.L., Tobi S., Wallace A.J., Perry M., Anup R., Lloyd S.K.W. (2020). Incidence of mosaicism in 1055 de novo NF2 cases: Much higher than previous estimates with high utility of next-generation sequencing. Anesth. Analg..

[77] Smith M.J., Bowers N.L., Banks C., Coates-Brown R., Morris K.A., Ewans L., Wilson M., Pinner J., Bhaskar S.S., Cammarata-Scalisi F. (2020). A deep intronic SMARCB1 variant associated with schwannomatosis. Clin. Genet..

[78] Nonaka D., Chiriboga L., Rubin B.P. (2008). Sox10: A Pan-Schwannian and Melanocytic Marker. Am. J. Surg. Pathol..

[79] Karamchandani J.R., Nielsen T.O., van de Rijn M., West R.B. (2012). Sox10 and S100 in the Diagnosis of Soft-tissue Neoplasms. Appl. Immunohistochem. Mol. Morphol..

[80] Jo V.Y., Fletcher C.D. (2017). SMARCB1/INI1 Loss in Epithelioid Schwannoma: A Clinicopathologic and Immunohistochemical Study of 65 Cases. Am. J. Surg. Pathol..

[81] Schaefer I.-M., Dong F., Garcia E.P., Fletcher C.D.M., Jo V.Y. (2019). Recurrent SMARCB1 Inactivation in Epithelioid Malignant Peripheral Nerve Sheath Tumors. Am. J. Surg. Pathol..

[82] Colman S.D., Williams C.A., Wallace M.R. (1995). Benign neurofibromas in type 1 neurofibromatosis (NF1) show somatic deletions of the NF1 gene. Nat. Genet..

[83] Klein C.J., Wu Y., Jentoft M.E., Mer G., Spinner R.J., Dyck P.J.B., Mauermann M.L. (2017). Genomic analysis reveals frequentTRAF7mutations in intraneural perineuriomas. Ann. Neurol..

[84] Carter J.M., Wu Y., Blessing M.M., Folpe A.L., Thorland E.C., Spinner R.J., Jentoft M.E., Wang C., Baheti S., Niu Z. (2018). Recurrent Genomic Alterations in Soft Tissue Perineuriomas. Am. J. Surg. Pathol..

[85] Brock J.E., Perez-Atayde A.R., Kozakewich H.P.W., Richkind K.E., Fletcher J.A., Vargas S.O. (2005). Cytogenetic Aberrations in Perineurioma: Variation with subtype. Am. J. Surg. Pathol..

[86] Ronellenfitsch M.W., Harter P.N., Kirchner M., Heining C., Hutter B., Gieldon L., Schittenhelm J., Schuhmann M.U., Tatagiba M., Marquardt G. (2020). Targetable ERBB2 mutations identified in neurofibroma/schwannoma hybrid nerve sheath tumors. J. Clin. Investig..

[87] Lee W., Teckie S., Wiesner T., Ran L., Granada C.N.P., Lin M., Zhu S., Cao Z., Liang Y., Sboner A. (2014). PRC2 is recurrently inactivated through EED or SUZ12 loss in malignant peripheral nerve sheath tumors. Nat. Genet..

[88] De Raedt T., Beert E., Pasmant E., Luscan A., Brems H., Ortonne N., Helin K., Hornick J.L., Mautner V., Kehrer-Sawatzki H. (2014). PRC2 loss amplifies Ras-driven transcription and confers sensitivity to BRD4-based therapies. Nature.

[89] Zhang M., Wang Y., Jones S., Sausen M., McMahon K., Sharma R., Wang Q., Belzberg A.J., Chaichana K., Gallia G.L. (2014). Somatic mutations of SUZ12 in malignant peripheral nerve sheath tumors. Nat. Genet..

[90] Pemov A., Hansen N.F., Sindiri S., Patidar R., Higham C.S., Dombi E., Miettinen M.M., Fetsch P., Brems H., Chandrasekharappa S.C. (2019). Low mutation burden and frequent loss of CDKN2A/B and SMARCA2, but not PRC2, define premalignant neurofibromatosis type 1–associated atypical neurofibromas. Neuro-Oncology.

[91] Prieto-Granada C.N., Wiesner T., Messina J.L., Jungbluth A.A., Chi P., Antonescu C.R. (2016). Loss of H3K27me3 Expression Is a Highly Sensitive Marker for Sporadic and Radiation-induced MPNST. Am. J. Surg. Pathol..

[92] Schaefer I.-M., Fletcher C.D., Hornick J.L. (2016). Loss of H3K27 trimethylation distinguishes malignant peripheral nerve sheath tumors from histologic mimics. Mod. Pathol..

[93] Bockmayr M., Körner M., Schweizer L., Schüller U. (2021). Cauda equina paragangliomas express HOXB13. Neuropathol. Appl. Neurobiol..

[94] Haller F., Moskalev E.A., Faucz F.R., Barthelmeß S., Wiemann S., Bieg M., Assié G., Bertherat J., Schaefer I.-M., Otto C. (2014). Aberrant DNA hypermethylation of SDHC: A novel mechanism of tumor development in Carney triad. Endocrine-Related Cancer.

[95] Letouzé E., Martinelli C., Loriot C., Burnichon N., Abermil N., Ottolenghi C., Janin M., Menara M., Nguyen A.T., Benit P. (2013). SDH Mutations Establish a Hypermethylator Phenotype in Paraganglioma. Cancer Cell.

[96] Walker F.O., Cartwright M.S., Alter K.E., Visser L.H., Hobson-Webb L.D., Padua L., Strakowski J.A., Preston D.C., Boon A.J., Axer H. (2018). Indications for neuromuscular ultrasound: Expert opinion and review of the literature. Clin. Neurophysiol..

[97] Hung E.H.Y., Griffith J.F., Ng A.W.H., Lee R.K.L., Lau D.T.Y., Leung J.C.S. (2014). Ultrasound of Musculoskeletal Soft-Tissue Tumors Superficial to the Investing Fascia. Am. J. Roentgenol..

[98] Tøttrup M., Eriksen J.D., Hellfritzsch M.B., Sørensen F.B., Baad-Hansen T. (2020). Diagnostic accuracy of ultrasound-guided core biopsy of peripheral nerve sheath tumors. J. Clin. Ultrasound.

[99] Pendleton C., Spinner R.J. (2020). Image-guided percutaneous biopsy of peripheral nerve tumors of indeterminate nature: Risks and benefits. Acta Neurochir..

[100] Grover S.B., Kundra R., Grover H., Gupta V., Gupta R. (2021). Imaging diagnosis of plexiform neurofibroma- unravelling the confounding features: A report of two cases. Radiol. Case Rep..

[101] Bruscella S., Alfieri A., de Bellis A., Rolando F., Covelli E.M., Manfredonia L., Orabona P., de Marinis P. (2021). Malignant Intracerebral Nerve Sheath Tumor Presenting with Intratumoral Hemorrhage. World Neurosurg..

[102] Tanaka M., Shibui S., Nomura K., Nakanishi Y., Hasegawa T., Hirose T., Le Fèvre C., Castelli J., Perrin C., Hénaux P. (2000). Malignant intracerebral nerve sheath tumor with intratumoral calcification. J. Neurosurg..

[103] Agarwal A., Chandra A., Jaipal U., Bagarhatta M., Mendiratta K., Goyal A., Kumar R., Mangalhara N. (2019). Can imaging be the new yardstick for diagnosing peripheral neuropathy?—A comparison between high resolution ultrasound and MR neurography with an approach to diagnosis. Insights Imaging.

[104] Noebauer-Huhmann I.M., Weber M.-A., Lalam R.K., Trattnig S., Bohndorf K., Vanhoenacker F., Tagliafico A., Van Rijswijk C., Vilanova J.C., Afonso P.D. (2015). Soft Tissue Tumors in Adults: ESSR-Approved Guidelines for Diagnostic Imaging. Semin. Musculoskelet. Radiol..

[105] Laffan E.E., Ngan B.-Y., Navarro O.M. (2009). Pediatric Soft-Tissue Tumors and Pseudotumors: MR Imaging Features with Pathologic Correlation: Part 2. Tumors of Fibroblastic/Myofibroblastic, So-called Fibrohistiocytic, Muscular, Lymphomatous, Neurogenic, Hair Matrix, and Uncertain Origin. Radiographics.

[106] Ahlawat S., Chhabra A., Blakely J. (2014). Magnetic Resonance Neurography of Peripheral Nerve Tumors and Tumorlike Conditions. Neuroimaging Clin. N. Am..

[107] Fisher S., Karri S., Ramzi A., Sharma R., Chhabra A., Soldatos T. (2015). Advanced MR Imaging of Peripheral Nerve Sheath Tumors Including Diffusion Imaging. Semin. Musculoskelet. Radiol..

[108] Chhabra A., Deshmukh S.D., Lutz A.M., Fritz J., Andreisek G., Sneag D.B., Subhawong T., Singer A.D., Wong P.K., Thakur U. (2022). Neuropathy Score Reporting and Data System: A Reporting Guideline for MRI of Peripheral Neuropathy with a Multicenter Validation Study. Am. J. Roentgenol..

[109] Chhabra A., Deshmukh S.D., Lutz A.M., Fritz J., Sneag D.B., Mogharrabi B., Guirguis M., Andreisek G., Xi Y., Ahlawat S. (2022). Neuropathy Score Reporting and Data System (NS-RADS): MRI Reporting Guideline of Peripheral Neuropathy Explained and Reviewed. Skelet. Radiol..

[110] Broski S.M., Johnson G., Howe B.M., Nathan M.A., Wenger D.E., Spinner R.J., Amrami K.K. (2016). Evaluation of 18F-FDG PET and MRI in differentiating benign and malignant peripheral nerve sheath tumors. Skelet. Radiol..

[111] Raad R.A., Lala S., Allen J.C., Babb J., Mitchell C.W., Franceschi A.M., Yohay K., Friedman K.P. (2018). Comparison of hybrid 18F-fluorodeoxyglucose positron emission tomography/magnetic resonance imaging and positron emission tomography/computed tomography for evaluation of peripheral nerve sheath tumors in patients with neurofibromatosis type 1. World J. Nucl. Med..

[112] Reinert C.P., Schuhmann M.U., Bender B., Gugel I., la Fougère C., Schäfer J., Gatidis S. (2019). Comprehensive anatomical and functional imaging in patients with type I neurofibromatosis using simultaneous FDG-PET/MRI. Eur. J. Nucl. Med..

[113] Bredella M.A., Torriani M., Hornicek F., Ouellette H.A., Plamer W.E., Williams Z., Fischman A.J., Plotkin S.R. (2007). Value of PET in the Assessment of Patients with Neurofibromatosis Type 1. Am. J. Roentgenol..

[114] Li C.-S., Huang G.-S., Wu H.-D., Chen W.-T., Shih L.-S., Lii J.-M., Duh S.-J., Chen R.-C., Tu H.-Y., Chan W.P. (2008). Differentiation of soft tissue benign and malignant peripheral nerve sheath tumors with magnetic resonance imaging. Clin. Imaging.

[115] Mautner V.F., Hartmann M., Kluwe L., Friedrich R.E., Fünsterer C. (2006). MRI growth patterns of plexiform neurofibromas in patients with neurofibromatosis type 1. Neuroradiology.

[116] Razek A.A.K.A., Ashmalla G.A. (2018). Assessment of paraspinal neurogenic tumors with diffusion-weighted MR imaging. Eur. Spine J..

[117] Fayad L.M., Wang X., Blakeley J.O., Durand D.J., Jacobs M.A., Demehri S., Subhawong T.K., Soldatos T., Barker P.B. (2014). Characterization of Peripheral Nerve Sheath Tumors with 3T Proton MR Spectroscopy. Am. J. Neuroradiol..

[118] Ogose A., Hotta T., Morita T., Higuchi T., Umezu H., Imaizumi S., Hatano H., Kawashima H., Gu W., Endo N. (2004). Diagnosis of Peripheral Nerve Sheath Tumors around the Pelvis. Jpn. J. Clin. Oncol..

[119] Lang S.-S., Zager E.L., Coyne T.M., Nangunoori R., Kneeland J.B., Nathanson K. (2012). Hybrid peripheral nerve sheath tumor: Case report. J. Neurosurg..

[120] Koeller K.K., Shih R.Y. (2019). Intradural Extramedullary Spinal Neoplasms: Radiologic-Pathologic Correlation. RadioGraphics.

[121] Nguyen R., Dombi E., Widemann B.C., Solomon J., Fuensterer C., Kluwe L., Friedman J.M., Mautner V.-F. (2012). Growth dynamics of plexiform neurofibromas: A retrospective cohort study of 201 patients with neurofibromatosis 1. Orphanet J. Rare Dis..

[122] Rubino F., Eichberg D.G., Shah A.H., Luther E.M., Lu V.M., Saad A.G., Kahn D., Komotar R.J., Ivan M.E. (2021). When “Peripheral” Becomes “Central”: Primary and Secondary Malignant Intracerebral Nerve Sheath Tumor: A Case Report and a Systematic Review. Neurosurgery.

[123] Le Fèvre C., Castelli J., Perrin C., Hénaux P.L., Noël G. (2016). Tumeurs malignes des gaines nerveuses périphériques intracérébrales métastatiques: À propos de deux cas et revue exhaustive des cas de la littérature. Cancer/Radiothérapie.

[124] Mackel C.E., Medeiros I., Moore B.E., Zhao Q., Jha R. (2021). Intracranial Malignant Peripheral Nerve Sheath Tumors Not Associated with a Cranial Nerve: Systematic Review and Illustrative Case. World Neurosurg..

[125] Kozić D., Nagulić M., Samardzić M., Ostojić J., Rasulić L., Cvetković-Dozić D. (2008). Intrapontine malignant nerve sheath tumor: MRI and MRS features. Acta Neurol. Belg..

[126] Ishimaru H., Morikawa M., Iwanaga S., Kaminogo M., Ochi M., Hayashi K. (2001). Differentiation between high-grade glioma and metastatic brain tumor using single-voxel proton MR spectroscopy. Eur. Radiol..

[127] Sundgren P., Annertz M., Englund E., Strömblad L.G., Holtås Ś. (1999). Paragangliomas of the spinal canal. Neuroradiology.

[128] Makhdoomi R., Nayil K., Santosh V. (2009). Primary Spinal Paragangliomas: A Review. Neurosurg. Q..

[129] Sharma A., Gaikwad S.B., Goyal M., Mishra N.K., Sharma M.C. (1998). Calcified filum terminale paraganglioma causing superficial siderosis. Am. J. Roentgenol..

[130] Schweizer L., Thierfelder F., Thomas C., Soschinski P., Suwala A., Stichel D., Wefers A.K., Wessels L., Misch M., Kim H.-Y. (2020). Molecular characterization of CNS paragangliomas identifies cauda equina paragangliomas as a distinct tumor entity. Acta Neuropathol..

[131] Klekamp J., Samii M. (1998). Surgery of Spinal Nerve Sheath Tumors with Special Reference to Neurofibromatosis. Neurosurgery.

[132] Robla-Costales J., Rodríguez-Aceves C., Martínez-Benia F., Socolovsky M. (2022). State of the Art and Advances in Peripheral Nerve Surgery. Adv. Tech. Stand. Neurosurg..

[133] Zipfel J., Al-Hariri M., Gugel I., Grimm A., Steger V., Ladurner R., Krimmel M., Tatagiba M., Schuhmann M. (2021). Surgical Management of Sporadic Peripheral Nerve Schwannomas in Adults: Indications and Outcome in a Single Center Cohort. Cancers.

[134] Pedro M.T., Antoniadis G., Scheuerle A., Pham M., Wirtz C.R., Koenig R.W. (2015). Intraoperative high-resolution ultrasound and contrast-enhanced ultrasound of peripheral nerve tumors and tumorlike lesions. Neurosurg. Focus.

[135] Gugel I., Grimm F., Tatagiba M., Schuhmann M.U., Zipfel J. (2022). Management of neurofibromatosis type 2 and schwannomatosis associated peripheral and intraspinal schwannomas: Influence of surgery, genetics, and localization. J. Neuro-Oncol..

[136] Siqueira M.G., Socolovsky M., Martins R.S., Robla-Costales J., Di Masi G., Heise C.O., Cosamalón J.G. (2013). Surgical treatment of typical peripheral schwannomas: The risk of new postoperative deficits. Acta Neurochir..

[137] Germano I.M., Sheehan J., Parish J., Atkins T., Asher A., Hadjipanayis C.G., Burri S.H., Green S., Olson J.J. (2018). Congress of Neurological Surgeons Systematic Review and Evidence-Based Guidelines on the Role of Radiosurgery and Radiation Therapy in the Management of Patients with Vestibular Schwannomas. Neurosurgery.

[138] Paldor I., Chen A.S., Kaye A.H. (2016). Growth rate of vestibular schwannoma. J. Clin. Neurosci..

[139] Hunter J.B., Francis D.O., O’Connell B.P., Kabagambe E.K., Bennett M.L., Wanna G.B., Rivas A., Thompson R.C., Haynes D.S. (2016). Single Institutional Experience with Observing 564 Vestibular Schwannomas: Factors Associated with Tumor Growth. Otol. Neurotol..

[140] Tveiten O.V., Carlson M.L., Goplen F., Vassbotn F., Link M.J., Lund-Johansen M. (2015). Long-term Auditory Symptoms in Patients with Sporadic Vestibular Schwannoma: An International Cross-Sectional Study. Neurosurgery.

[141] Goldbrunner R., Weller M., Regis J., Lund-Johansen M., Stavrinou P., Reuss D., Evans D.G., Lefranc F., Sallabanda K., Falini A. (2019). EANO guideline on the diagnosis and treatment of vestibular schwannoma. Neuro-Oncology.

[142] Hadjipanayis C.G., Carlson M.L., Link M.J., Rayan T.A., Parish J., Atkins T., Asher A.L., Dunn I.F., Corrales C.E., Van Gompel J.J. (2018). Congress of Neurological Surgeons Systematic Review and Evidence-Based Guidelines on Surgical Resection for the Treatment of Patients with Vestibular Schwannomas. Neurosurgery.

[143] Ferrari A., Casanova M., Bisogno G., Mattke A., Meazza C., Gandola L., Sotti G., Cecchetto G., Harms D., Koscielniak E. (2002). Clear cell sarcoma of tendons and aponeuroses in pediatric patients: A report from the Italian and German Soft Tissue Sarcoma Cooperative Group. Cancer.

[144] Gachiani J., Kim D., Nelson A., Kline D. (2007). Surgical management of malignant peripheral nerve sheath tumors. Neurosurg. Focus.

[145] Kahn J., Gillespie A., Tsokos M., Ondos J., Dombi E., Camphausen K., Widemann B.C., Kaushal A. (2014). Radiation Therapy in Management of Sporadic and Neurofibromatosis Type 1-Associated Malignant Peripheral Nerve Sheath Tumors. Front. Oncol..

[146] Martin E., Coert J.H., Flucke U.E., Slooff W.-B.M., Ho V.K., van der Graaf W.T., van Dalen T., van de Sande M.A., van Houdt W.J., Grünhagen D.J. (2020). A nationwide cohort study on treatment and survival in patients with malignant peripheral nerve sheath tumours. Eur. J. Cancer.

[147] Spunt S.L., Million L., Chi Y.-Y., Anderson J., Tian J., Hibbitts E., Coffin C., McCarville M.B., Randall R.L., Parham D.M. (2020). A risk-based treatment strategy for non-rhabdomyosarcoma soft-tissue sarcomas in patients younger than 30 years (ARST0332): A Children’s Oncology Group prospective study. Lancet Oncol..

[148] Myrseth E., Møller P., Pedersen P.-H., Lund-Johansen M. (2009). Vestibular schwannoma: Surgery or gamma knife radiosurgery? A prospective, nonrandomized study. Neurosurgery.

[149] Régis J., Pellet W., Delsanti C., Dufour H., Roche P.H., Thomassin J.M., Zanaret M., Peragut J.C. (2002). Functional outcome after gamma knife surgery or microsurgery for vestibular schwannomas. J. Neurosurg..

[150] Karpinos M., Teh B.S., Zeck O., Carpenter L., Phan C., Mai W.-Y., Lu H.H., Chiu J., Butler E., Gormley W.B. (2002). Treatment of acoustic neuroma: Stereotactic radiosurgery vs. microsurgery. Int. J. Radiat. Oncol..

[151] Pollock B.E., Driscoll C.L., Foote R.L., Link M.J., Gorman D.A., Bauch C.D., Mandrekar J.N., Krecke K.N., Johnson C.H. (2006). Patient Outcomes After Vestibular Schwannoma Management: A Prospective Comparison of Microsurgical Resection and Stereotactic Radiosurgery. Neurosurgery.

[152] Carlson M.L., Vivas E.X., McCracken D.J., Sweeney A.D., Neff B.A., Shepard N.T., Olson J.J. (2018). Congress of Neurological Surgeons Systematic Review and Evidence-Based Guidelines on Hearing Preservation Outcomes in Patients with Sporadic Vestibular Schwannomas. Neurosurgery.

[153] Tsao M.N., Sahgal A., Xu W., De Salles A., Hayashi M., Levivier M., Ma L., Martinez R., Régis J., Ryu S. (2017). Stereotactic radiosurgery for vestibular schwannoma: International Stereotactic Radiosurgery Society (ISRS) Practice Guideline. J. Radiosurg. SBRT.

[154] Huy P.T.B. (2014). Radiotherapy for glomus jugulare paraganglioma. Eur. Ann. Otorhinolaryngol. Head Neck Dis..

[155] Plotkin S.R., Merker V.L., Halpin C., Jennings D., McKenna M.J., Harris G.J., Barker F.G.I. (2012). Bevacizumab for Progressive Vestibular Schwannoma in Neurofibromatosis Type 2: A retrospective review of 31 patients. Otol. Neurotol..

[156] Blakeley J.O., Ye X., Duda D.G., Halpin C.F., Bergner A.L., Muzikansky A., Merker V., Gerstner E.R., Fayad L.M., Ahlawat S. (2016). Efficacy and Biomarker Study of Bevacizumab for Hearing Loss Resulting from Neurofibromatosis Type 2–Associated Vestibular Schwannomas. J. Clin. Oncol..

[157] Robertson K.A., Nalepa G., Yang F.-C., Bowers D.C., Ho C.Y., Hutchins G.D., Croop J.M., Vik T.A., Denne S.C., Parada L.F. (2012). Imatinib mesylate for plexiform neurofibromas in patients with neurofibromatosis type 1: A phase 2 trial. Lancet Oncol..

[158] Widemann B.C., Dombi E., Gillespie A., Wolters P.L., Belasco J., Goldman S., Korf B.R., Solomon J., Martin S., Salzer W. (2014). Phase 2 randomized, flexible crossover, double-blinded, placebo-controlled trial of the farnesyltransferase inhibitor tipifarnib in children and young adults with neurofibromatosis type 1 and progressive plexiform neurofibromas. Neuro-Oncology.

[159] Widemann B.C., Babovic-Vuksanovic D., Dombi E., Wolters P.L., Goldman S., Martin S., Goodwin A., Goodspeed W., Kieran M.W., Cohen B. (2014). Phase II trial of pirfenidone in children and young adults with neurofibromatosis type 1 and progressive plexiform neurofibromas. Pediatr. Blood Cancer.

[160] Weiss B., Widemann B.C., Wolters P., Dombi E., Vinks A.A., Cantor A., Korf B., Perentesis J., Gutmann D.H., Schorry E. (2013). Sirolimus for non-progressive NF1-associated plexiform neurofibromas: An NF clinical trials consortium phase II study. Pediatr. Blood Cancer.

[161] Weiss B., Widemann B.C., Wolters P., Dombi E., Vinks A., Cantor A., Perentesis J., Schorry E., Ullrich N., Gutmann D.H. (2015). Sirolimus for progressive neurofibromatosis type 1-associated plexiform neurofibromas: A Neurofibromatosis Clinical Trials Consortium phase II study. Neuro-Oncology.

[162] Jakacki R.I., Dombi E., Steinberg S.M., Goldman S., Kieran M.W., Ullrich N.J., Pollack I.F., Goodwin A., Manley P.E., Fangusaro J. (2017). Phase II trial of pegylated interferon alfa-2b in young patients with neurofibromatosis type 1 and unresectable plexiform neurofibromas. Neuro-Oncology.

[163] Zehou O., Ferkal S., Brugieres P., Barbarot S., Bastuji-Garin S., Combemale P., Valeyrie-Allanore L., Sbidian E., Wolkenstein P. (2019). Absence of Efficacy of Everolimus in Neurofibromatosis 1-Related Plexiform Neurofibromas: Results from a Phase 2a Trial. J. Investig. Dermatol..

[164] Gross A.M., Dombi E., Widemann B.C. (2020). Current status of MEK inhibitors in the treatment of plexiform neurofibromas. Child’s Nerv. Syst..

[165] Dombi E., Baldwin A., Marcus L.J., Fisher M.J., Weiss B., Kim A., Whitcomb P., Martin S., Aschbacher-Smith L.E., Rizvi T.A. (2016). Activity of Selumetinib in Neurofibromatosis Type 1–Related Plexiform Neurofibromas. N. Engl. J. Med..

[166] Gross A.M., Wolters P.L., Dombi E., Baldwin A., Whitcomb P., Fisher M.J., Weiss B., Kim A., Bornhorst M., Shah A.C. (2020). Selumetinib in Children with Inoperable Plexiform Neurofibromas. N. Engl. J. Med..

[167] Gross A.M., Glassberg B., Wolters P.L., Dombi E., Baldwin A., Fisher M.J., Kim A.R., Bornhorst M., Weiss B.D., Blakeley J.O. (2022). Selumetinib in children with neurofibromatosis type 1 and asymptomatic inoperable plexiform neurofibroma at risk for developing tumor-related morbidity. Neuro-Oncology.

[168] Jackson S., Baker E.H., Gross A.M., Whitcomb P., Baldwin A., Derdak J., Tibery C., Desanto J., Carbonell A., Yohay K. (2020). The MEK inhibitor selumetinib reduces spinal neurofibroma burden in patients with NF1 and plexiform neurofibromas. Neuro-Oncol. Adv..

[169] Coyne G.H.O., Gross A.M., Dombi E., Tibery C., Carbonell A., Takebe N., Derdak J., Pichard D., Srivastava A.K., Herrick W. (2020). Phase II trial of the MEK 1/2 inhibitor selumetinib (AZD6244, ARRY-142886 Hydrogen Sulfate) in adults with neurofibromatosis type 1 (NF1) and inoperable plexiform neurofibromas (PN). J. Clin. Oncol..

[170] Santo V.E., Passos J., Nzwalo H., Carvalho I., Santos F., Martins C., Salgado L., Silva C.E., Vinhais S., Vilares M. (2020). Selumetinib for plexiform neurofibromas in neurofibromatosis type 1: A single-institution experience. J. Neuro-Oncol..

[171] Weiss B.D., Wolters P.L., Plotkin S.R., Widemann B.C., Tonsgard J.H., Blakeley J., Allen J.C., Schorry E., Korf B., Robison N.J. (2021). NF106: A Neurofibromatosis Clinical Trials Consortium Phase II Trial of the MEK Inhibitor Mirdametinib (PD-0325901) in Adolescents and Adults with NF1-Related Plexiform Neurofibromas. J. Clin. Oncol..

[172] McCowage G.B., Mueller S., Pratilas C.A., Hargrave D.R., Moertel C.L., Whitlock J., Fox E., Hingorani P., Russo M.W., Dasgupta K. (2018). Trametinib in pediatric patients with neurofibromatosis type 1 (NF-1)–associated plexiform neurofibroma: A phase I/IIa study. J. Clin. Oncol..

[173] Wang D., Ge L., Guo Z., Li Y., Zhu B., Wang W., Wei C., Li Q., Wang Z. (2022). Efficacy and Safety of Trametinib in Neurofibromatosis Type 1-Associated Plexiform Neurofibroma and Low-Grade Glioma: A Systematic Review and Meta-Analysis. Pharmaceuticals.

[174] Mueller S., Reddy A.T., Dombi E., Allen J., Packer R., Clapp W., Goldman S., Schorry E., Tonsgard J., Blakeley J. (2020). MEK inhibitor binimetinib shows clinical activity in children with neurofibromatosis type 1-associated plexiform neurofibromas: A report from PNOC and the NF clinical trials consortium. Neuro Oncol..

[175] Fisher M.J., Shih C.-S., Rhodes S.D., Armstrong A.E., Wolters P.L., Dombi E., Zhang C., Angus S.P., Johnson G.L., Packer R.J. (2021). Cabozantinib for neurofibromatosis type 1–related plexiform neurofibromas: A phase 2 trial. Nat. Med..

[176] Nutakki K., Varni J.W., Swigonski N.L. (2018). PedsQL Neurofibromatosis Type 1 Module for children, adolescents and young adults: Feasibility, reliability, and validity. J. Neuro-Oncol..

[177] Wang J., Pollard K., Calizo A., Pratilas C.A. (2021). Activation of Receptor Tyrosine Kinases Mediates Acquired Resistance to MEK Inhibition in Malignant Peripheral Nerve Sheath Tumors. Cancer Res.

[178] Cai Z., Tang X., Liang H., Yang R., Yan T., Guo W. (2020). Prognosis and risk factors for malignant peripheral nerve sheath tumor: A systematic review and meta-analysis. World J. Surg. Oncol..

[179] Yan P., Huang R., Hu P., Liu F., Zhu X., Hu P., Yin H., Zhang J., Meng T., Huang Z. (2019). Nomograms for predicting the overall and cause-specific survival in patients with malignant peripheral nerve sheath tumor: A population-based study. J. Neuro-Oncol..

[180] Judson I., Verweij J., Gelderblom H., Hartmann J.T., Schöffski P., Blay J.-Y., Kerst J.M., Sufliarsky J., Whelan J., Hohenberger P. (2014). Doxorubicin alone versus intensified doxorubicin plus ifosfamide for first-line treatment of advanced or metastatic soft-tissue sarcoma: A randomised controlled phase 3 trial. Lancet Oncol..

[181] Skotheim R.I., Kallioniemi A., Bjerkhagen B., Mertens F., Brekke H.R., Monni O., Mousses S., Mandahl N., Sœter G., Nesland J.M. (2003). Topoisomerase-IIα Is Upregulated in Malignant Peripheral Nerve Sheath Tumors and Associated with Clinical Outcome. J. Clin. Oncol..

[182] Higham C.S., Steinberg S.M., Dombi E., Perry A., Helman L.J., Schuetze S.M., Ludwig J.A., Staddon A., Milhem M., Rushing D. (2017). SARC006: Phase II Trial of Chemotherapy in Sporadic and Neurofibromatosis Type 1 Associated Chemotherapy-Naive Malignant Peripheral Nerve Sheath Tumors. Sarcoma.

[183] Steins M.B., Serve H., Zühlsdorf M., Senninger N., Semik M., Berdel W.E. (2002). Carboplatin/etoposide induces remission of metasta-sised malignant peripheral nerve tumours (malignant schwannoma) refractory to first-line therapy. Oncol Rep..

[184] Leu K.M., Ostruszka L.J., Shewach D., Zalupski M., Sondak V., Biermann J.S., Lee J.S.-J., Couwlier C., Palazzolo K., Baker L.H. (2004). Laboratory and Clinical Evidence of Synergistic Cytotoxicity of Sequential Treament with Gemcitabine Followed by Docetaxel in the Treatment of Sarcoma. J. Clin. Oncol..

[185] Takahashi M., Komine K., Imai H., Okada Y., Saijo K., Takahashi M., Shirota H., Ohori H., Takahashi S., Chiba N. (2017). Efficacy and safety of gemcitabine plus docetaxel in Japanese patients with unresectable or recurrent bone and soft tissue sarcoma: Results from a single-institutional analysis. PLoS ONE.

[186] Moretti V.M., Crawford E.A., Staddon A.P., Lackman R.D., Ogilvie C.M. (2011). Early Outcomes for Malignant Peripheral Nerve Sheath Tumor Treated with Chemotherapy. Am. J. Clin. Oncol..

[187] Kaplan H.G., Rostad S., Ross J.S., Ali S.M., Millis S.Z. (2018). Genomic Profiling in Patients with Malignant Peripheral Nerve Sheath Tumors Reveals Multiple Pathways with Targetable Mutations. J. Natl. Compr. Cancer Netw..

[188] Nagabushan S., Lau L.M.S., Barahona P., Wong M., Sherstyuk A., Marshall G.M., Tyrrell V., Wegner E.A., Ekert P.G., Cowley M.J. (2021). Efficacy of MEK inhibition in a recurrent malignant peripheral nerve sheath tumor. NPJ Precis. Oncol..

[189] Grit J.L., Pridgeon M.G., Essenburg C.J., Wolfrum E., Madaj Z.B., Turner L., Wulfkuhle J., Iii E.F.P., Graveel C.R., Steensma M.R. (2020). Kinome Profiling of NF1-Related MPNSTs in Response to Kinase Inhibition and Doxorubicin Reveals Therapeutic Vulnerabilities. Genes.

[190] Maki R.G., D’Adamo D.R., Keohan M.L., Saulle M., Schuetze S.M., Undevia S.D., Livingston M.B., Cooney M.M., Hensley M.L., Mita M.M. (2009). Phase II Study of Sorafenib in Patients with Metastatic or Recurrent Sarcomas. J. Clin. Oncol..

[191] González-Muñoz T., Kim A., Ratner N., Peinado H. (2022). The Need for New Treatments Targeting MPNST: The Potential of Strategies Combining MEK Inhibitors with Antiangiogenic Agents. Clin. Cancer Res..

[192] Widemann B.C., Lu Y., Reinke D., Okuno S.H., Meyer C.F., Cote G.M., Chugh R., Milhem M., Hirbe A.C., Kim A. (2019). Targeting Sporadic and Neurofibromatosis Type 1 (NF1) Related Refractory Malignant Peripheral Nerve Sheath Tumors (MPNST) in a Phase II Study of Everolimus in Combination with Bevacizumab (SARC016). Sarcoma.

[193] Kim A., Lu Y., Okuno S.H., Reinke D., Maertens O., Perentesis J., Basu M., Wolters P.L., De Raedt T., Chawla S. (2020). Targeting Refractory Sarcomas and Malignant Peripheral Nerve Sheath Tumors in a Phase I/II Study of Sirolimus in Combination with Ganetespib (SARC023). Sarcoma.

[194] Chugh R., Wathen J.K., Maki R.G., Benjamin R.S., Patel S.R., Myers P.A., Priebat D.A., Reinke D.K., Thomas D.G., Keohan M.L. (2009). Phase II Multicenter Trial of Imatinib in 10 Histologic Subtypes of Sarcoma Using a Bayesian Hierarchical Statistical Model. J. Clin. Oncol..

[195] Schuetze S.M., Wathen J.K., Lucas D.R., Choy E., Samuels B.L., Staddon A.P., Ganjoo K.N., von Mehren M., Chow W.A., Loeb D.M. (2015). SARC009: Phase 2 study of dasatinib in patients with previously treated, high-grade, advanced sarcoma. Cancer.

[196] Manji G.A., Van Tine B.A., Lee S.M., Raufi A.G., Pellicciotta I., Hirbe A.C., Pradhan J., Chen A., Rabadan R., Schwartz G.K. (2021). A Phase I Study of the Combination of Pexidartinib and Sirolimus to Target Tumor-Associated Macrophages in Unresectable Sarcoma and Malignant Peripheral Nerve Sheath Tumors. Clin. Cancer Res..

[197] Nishida Y., Urakawa H., Nakayama R., Kobayashi E., Ozaki T., Ae K., Matsumoto Y., Tsuchiya H., Goto T., Hiraga H. (2021). PhaseIIclinical trial of pazopanib for patients with unresectable or metastatic malignant peripheral nerve sheath tumors. Int. J. Cancer.

[198] Albritton K.H., Rankin C., Coffin C.M., Ratner N., Budd G.T., Schuetze S.M., Randall R.L., DeClue J.E., Borden E.C. (2006). Phase II study of erlotinib in metastatic or unresectable malignant peripheral nerve sheath tumors (MPNST). J. Clin. Oncol..

[199] Dickson M.A., Mahoney M.R., Tap W.D., D’Angelo S.P., Keohan M.L., Van Tine B.A., Agulnik M., Horvath L.E., Nair J.S., Schwartz G.K. (2016). Phase II study of MLN8237 (Alisertib) in advanced/metastatic sarcoma. Ann. Oncol..

[200] Al-Ezzi E., Gounder M., Watson G., Mazzocca A., D’Angelo S.P., Bravetti J., Wang H., Razak A.A., Vincenzi B. (2021). Selinexor, a First in Class, Nuclear Export Inhibitor for the Treatment of Advanced Malignant Peripheral Nerve Sheath Tumor. Oncologist.

[201] Kobayashi H., Makise N., Shinozaki-Ushiku A., Zhang L., Ishibashi Y., Ikegami M., Tsuda Y., Kohsaka S., Ushiku T., Oda K. (2022). Dramatic response to entrectinib in a patient with malignant peripheral nerve sheath tumor harboring novel SNRNP70-NTRK3 fusion gene. Genes Chromosom. Cancer.

[202] Özdemir B.C., Bohanes P., Bisig B., Missiaglia E., Tsantoulis P., Coukos G., Montemurro M., Homicsko K., Michielin O. (2019). Deep Response to Anti-PD-1 Therapy of Metastatic Neurofibromatosis Type 1-Associated Malignant Peripheral Nerve Sheath Tumor wth CD274/PD-L1 Amplification. JCO Precis. Oncol..

[203] Davis L.E., Nicholls L.A., Babiker H.M., Liau J., Mahadevan D. (2019). PD-1 Inhibition Achieves a Complete Metabolic Response in a Patient with Malignant Peripheral Nerve Sheath Tumor. Cancer Immunol. Res..

